# Characterization of dynamic compliance of the respiratory system in healthy anesthetized dogs

**DOI:** 10.3389/fvets.2024.1490494

**Published:** 2024-11-28

**Authors:** Mathieu Raillard, Martina Mosing, Anthea Raisis, Adam Auckburally, Georgina Beaumont, Frances Downing, Charlotte Heselton, Paul MacFarlane, Karine Portier, Josephine Robertson, Joao Henrique Neves Soares, Barbara Steblaj, Elliot Wringe, Olivier L. Levionnois

**Affiliations:** ^1^School of Veterinary Medicine, College of Environmental and Life Sciences, Murdoch University, Murdoch, WA, Australia; ^2^Anaesthesiology and Perioperative Intensive Care, Clinical Department for Small Animals and Horses, Veterinary University Vienna, Vienna, Austria; ^3^Southern Counties Veterinary Specialists, Ringwood, United Kingdom; ^4^Manchester Veterinary Specialists, Manchester, United Kingdom; ^5^Davies Veterinary Specialists, Herts, United Kingdom; ^6^Langford Vets, University of Bristol, Langford, United Kingdom; ^7^VetAgro Sup (Campus Vétérinaire), Centre de Recherche et de Formation en Algologie Comparée (CREFAC), University of Lyon, Marcy l'Etoile, France; ^8^Université Claude Bernard Lyon, Centre de Recherche en Neurosciences de Lyon, INSERM, CRNL U1028 UMR5292, Lyon, France; ^9^Small Animal Hospital, University of Glasgow, Glasgow, United Kingdom; ^10^Department of Surgical and Radiological Sciences, School of Veterinary Medicine, University of California, Davis, Davis, CA, United States; ^11^Section Anaesthesiology, Department of Diagnostics and Clinical Sciences, Vetsuisse Faculty, University of Zürich, Zürich, Switzerland; ^12^Division of Anaesthesiology and Pain Therapy, Vetsuisse Faculty, University of Bern, Bern, Switzerland

**Keywords:** anesthesia, compliance, dogs, dynamic compliance, monitoring, respiratory mechanics, spirometry, ventilation

## Abstract

**Introduction:**

In clinical practice, evaluating dynamic compliance of the respiratory system (C_dyn_) could provide valuable insights into respiratory mechanics. Reference values of C_dyn_ based on body weight have been reported, but various factors may affect them and the evidence is scanty. This study aimed to establish a reference interval for C_dyn_ and identify associated variables.

**Methods:**

Data were collected from 515 client-owned dogs requiring anesthesia, excluding those with lower airway disease. The dogs were anesthetized, the tracheas intubated, and lungs ventilated at clinicians' discretion across 11 centers in six countries, with no restrictions on anesthesia protocols or ventilation settings, except avoiding inspiratory pauses. Three C_dyn_ measurements from three consecutive breaths per dog were recorded using a standardized form, which also documented factors affecting C_dyn_ identified through literature and an online survey. Various spirometry technologies were used. The substantial variance in C_dyn_ measurements led to a comprehensive analysis using a multiple linear regression model. Multicollinearity (variables highly correlated with each other) was addressed by investigating, transforming, or excluding factors. Initial simple linear regression assessed each variable's individual effect on C_dyn_, followed by a multiple linear regression model constructed via stepwise forward selection and backward elimination.

**Results:**

The best-fitting model identified a linear relationship between C_dyn_ and body mass when the following conditions were met: high BCS (Body Condition Score), orotracheal tubes <7% smaller than predicted, the use of a D-lite flow sensor, and the absence of a high FIO2 (>80%) exposure for more than 10 minutes before C_dyn_ measurement. In cases where these conditions were not met, additional factors needed to be incorporated into the model. Low (1/9, 2/9, 3/9) and medium (4/9, 5/9) BCS, an orotracheal tube of the predicted size or larger and longer inspiratory times were associated with increased C_dyn_. The use of alternative spirometry sensors, including Ped-lite, or prolonged exposure to high FIO_2_ levels resulted in decreased C_dyn_.

**Conclusion and clinical relevance:**

Establishing a reference interval for C_dyn_ proved challenging. A single reference interval may be misleading or unhelpful in clinical practice. Nevertheless, this study offers valuable insights into the factors affecting C_dyn_ in healthy anesthetized dogs, which should be considered in clinical assessments.

## 1 Introduction

Spirometry is a common monitoring modality used in clinical practice in anesthetized dogs (1). The pressure-volume relationship of the respiratory system (P-V) is fundamental for assessing its mechanics during mechanical ventilation (2). Compliance is defined as the change in lung volume per unit change in pressure gradient; it may be measured for lung, thoracic cage, or both [i.e., respiratory system; (3)]. Compliance plays a pivotal role in understanding respiratory physiology in veterinary anesthesia, particularly when dogs are mechanically ventilated. It is commonly displayed by modern monitors, and generally calculated dividing the expired gas volume by the change in the airway pressure (plateau pressure—positive end expiratory pressure). Various types of compliance, including dynamic compliance (C_dyn_), static compliance (C_stat_), and quasi-static compliance, have been utilized to evaluate respiratory system function in response to interventions and different influencing factors (4–7). Both C_stat_ and quasi-static compliance require an inspiratory pause. Quasi-static compliance, obtained with inspiratory pauses of variable durations below 3 s, did not provide accurate C_stat_ values in healthy dogs (8). C_dyn_ offers a real-time assessment of respiratory performance during mechanical ventilation, enabling the early detection of changes in airway resistance—such as bronchospasm, secretions, and tube kinking—as well as alterations in the elastic component (9).

Despite decades of research on P-V curves, our comprehensive understanding of the P-V relationship remains incomplete (2). C_dyn_ can be assessed as a loop or as a numerical value. A flatter loop suggests lower compliance, potentially indicating stiffening of lung tissue or the chest wall, whereas a steeper loop indicates higher compliance (10). Veterinary anesthetists typically use the shape and steepness of the P-V loop independently of numerical values for C_dyn_ to guide their clinical decisions (1). The shape and steepness of P-V loops are greatly influenced by the scale of the P-V graph displayed. The axes scales can be altered either manually or automatically by devices. Therefore, interpreting C_dyn_ based on the steepness of a P-V curve through subjective visual observation may lead to incorrect conclusions, particularly given the existing gap in the literature concerning C_dyn_ usual values. Creating a quantitative reference range for C_dyn_ may assist clinical interpretation of changes in lung mechanics.

However, developing a reference range is likely to be challenging due to the different variables influencing compliance that have been identified in dogs. Body mass is a logical variable due to its impact on tidal volume (V_T_), as described by Stahl (11), and indicated in equations reported by Asorey et al. (12) and Bradbrook et al. (13). Additionally, the influence of body position on C_dyn_ (13) and inspiratory time (14) have been demonstrated. These variables underscore the multifaceted nature of compliance measurements and highlight the need for establishing reference intervals specific to the canine population.

The goals of this prospective, exploratory study were to establish a reference interval for C_dyn_ in anesthetized dogs and to identify the variables impacting C_dyn_.

## 2 Materials and methods

### 2.1 Centers included

This study was carried out in 11 centers located in five countries: Australia, France, Switzerland, The United Kingdom, and The United States of America. An overall ethical approval was granted by the Ethical Review Group of the Association of Veterinary Anesthetists (2019-007). This approval was sufficient for the following centers: Southern Counties Veterinary Specialists, Davies Veterinary Specialists, Manchester Veterinary Specialists, Small Animal Hospital of the University of Glasgow, University of California and VetAgro Sup. The project was further approved by the relevant local ethical committees at Murdoch University (R3186/19), the University of Bristol (VIN/18/032), the University of Sydney (2019/1617), and Vetsuisse Faculty Bern & Zürich (BE78/18).

### 2.2 Case selection by centers

Cases were collected between July 2019 and November 2023. If the hospital's admission or anesthesia consent forms lacked authorization to record clinical data, an informed owner consent form was provided by study organizers.

Based on a full physical examination, client-owned dogs with an ASA classification of I and II (American Society of Anesthesiologists) undergoing anesthesia according to their clinical condition were recruited at the discretion of the responsible anesthetists in each center. The exclusion criterion included: history of lung disease, known lower respiratory tract disease and any intra-anesthetic complication (e.g., hypotension, hypoxaemia) at the time of the measurement. The tracheas of the dogs had to be intubated (orotracheal intubation) and the lungs mechanically ventilated for them to be included in the study.

### 2.3 Anesthetic management and ventilation monitoring

A variety of spirometry technologies and monitors is commonly used in veterinary anesthesia (1) and different models were available in the centers involved in the study. Therefore, the equipment type was not restricted.

The original plan was to conduct calibration checks on the monitors just before beginning data collection at each center. However, due to significant delays caused by the COVID-19 pandemic, data was collected without considering the calibration status of the monitors.

Cases were managed according to normal clinical practice in the hospitals, without any special adaptation for the study. Dogs were anesthetized, monitored, and ventilated as appropriate, with no restrictions on anesthesia protocol or ventilation settings, except a request not to use any inspiratory pause.

Cases were performed by any anesthetist at any of the centers, under the supervision of a main local coordinator. Whenever possible, the measurements of C_dyn_ were to be recorded within the first hour of establishing controlled mechanical ventilation. Immediately prior to recording C_dyn_, the anesthetists ensured leaks were absent and that there was no dog-ventilator asynchrony. Measurements were postponed if a leak was detected based on evaluating closure of the P-V loop, the difference between inspiratory and expiratory volumes, inadequate airway pressure and flow, and capnography waveforms in relation to chest wall movements. For each subject, measurements of C_dyn_ from three consecutive breaths were recorded.

### 2.4 Data collection form

Factors likely to impact on C_dyn_, identified from previous literature and through an online survey, were recorded. These factors included:

- Body mass (in kg),
- Internal diameter (in mm) and length (in cm) of the orotracheal tubes,
- Age (in months),
- Breed,
- Body Condition Score (BCS) scored out of 9 (https://wsava.org/wp-content/uploads/2020/01/Body-Condition-Score-Dog.pdf),
- Morphology (brachymorphic, dolichomorphic, mesomorphic; subjective evaluation),
- Positioning (dorsal, lateral, or sternal recumbency) with or without Trendelenburg (No, Trendelenburg or reverse Trendelenburg),
- Breathing system (circle or Mapleson),
- Spirometry sensor/monitor used (classified for analysis as: D-Lite, Pedi-Lite, other),
- The use of Heat and Moisture Exchangers (HMEs, present or absent between the spirometry sensor or the breathing system and the orotracheal tube),
- The inspired fraction of oxygen (FIO_2_, %),
- Whether or not a FIO_2_ above 80% was maintained for 10 minutes or more before C_dyn_ determination (yes or no),
- The time elapsed between the induction of anesthesia and the measurements (in minutes),
- The ventilation mode (volume-controlled or pressure-controlled ventilation),
- The positive end-expiratory pressure set at the time of the measurements (PEEP, in cmH_2_O),
- The inspiratory time (in seconds, calculated from the respiratory rate and the inspiratory to expiratory ratio setting),
- The drugs administered before the measurements (yes or no, for alpha-2 adrenergic receptor agonists, acepromazine, alfaxalone, anti-muscarinic, benzodiazepines, butorphanol, intravenous lidocaine, isoflurane, ketamine, mu-agonist opioids, neuromuscular blocking agents, propofol, and sevoflurane),
- The administration of a locoregional block, an epidural or a spinal injection (yes or no).

Some variables were recorded only to confirm that the individual cases were not subject to any obvious anesthetic complications at the time of measurement (e.g., hemoglobin saturation <94%, end tidal CO_2_ > 60 mmHg, Mean Arterial Pressure <60 mmHg), but were not included in the statistical analysis.

**Figure 1:**
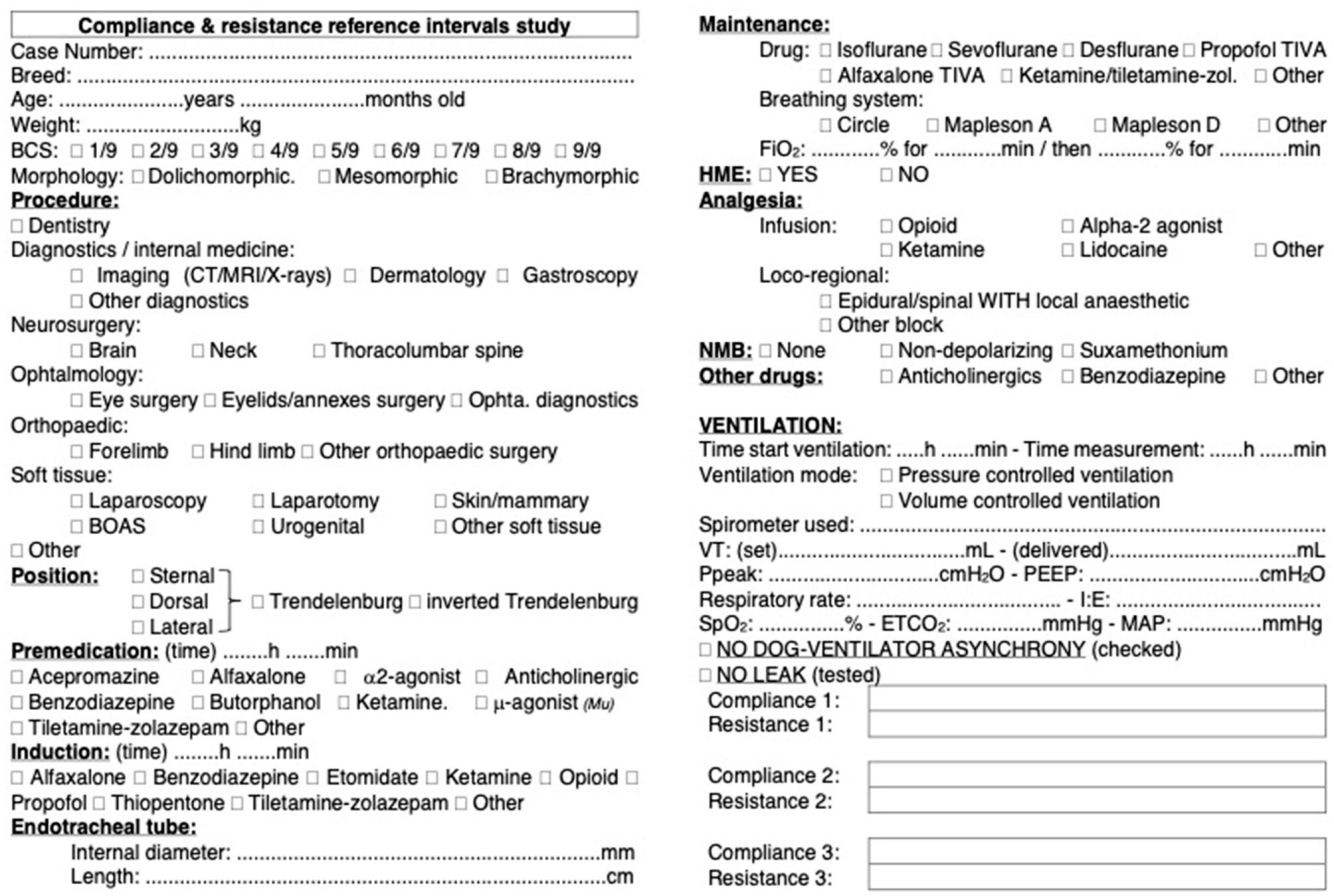
**Data collection form for dynamic compliance (C**_**dyn**_**) of the respiratory system in dogs**. Standardized data collection form used across 11 centers in six countries to assess C_dyn_ in a cohort of 515 dogs. The form includes fields for recording variables that were identified as potentially influencing C_dyn_. Each center adhered to the same protocol for data collection to ensure consistency and reliability of the data across different locations.

A standard data collection form was used in each case. The data collection form was trialed by three clinical anesthetists before the start of the study to ensure it was comprehensive and easily filled. The form is presented in Figure 1. The data obtained from the various centers was compiled in an Excel file.

### 2.5 Data curation

Data was excluded from cases with more than 15% variation in C_dyn_ across the three measurements. A 15% threshold was arbitrarily chosen by the authors as it accounted for potential variability in the precision of the instruments.

Cases containing missing or obviously incorrect values within selected variables were flagged. Data from those cases was excluded when variables were analyzed independently. If a factor was included in the final multiple linear regression model, data from the cases with missing or incorrect values were excluded. For each test, the total number of cases included in the analysis is reported.

In addition, categories (i.e., factors likely to impact C_dyn_) that appeared in fewer than 25 cases (an arbitrary number) were not considered in the analysis.

### 2.6 Number of animals

The sample size was determined to ensure adequate statistical power for evaluating the reference interval of C_dyn_ in dogs under anesthesia and determining the possible influence of 20 variables and 21 drugs and loco regional techniques. We integrated previously published data on compliance variability to estimate a standard deviation (σ) of 6.6 mL/cmH_2_O (13). Using this information, along with a desired precision (δ) of ±5 mL/cmH_2_O, a significance level (α) of 0.05, and a desired power (1–β) of 0.80, the required sample size was calculated. Considering the multicentre nature of the study, an arbitrary design effect of 1.5 was applied to adjust the sample size calculation, accounting for potential variability between centers. The resulting required sample size, considering both multicentre adjustment and compliance standard deviation, was determined to be ~303 dogs. Since the study was non-invasive, data collection was limited in time rather than once the predetermined number of cases was reached. This approach aimed to maximize the chances of obtaining sufficient representation for each evaluated variable.

### 2.7 Statistical analysis

Statistical analysis was performed using R version 4.3.2 (15). The “pwr” package was used to perform the sample size calculation. The package “readxl” was used to import the data in R (16). The package “ggplot2” was used for graphical representations, unless stated otherwise (17).

The average of the three consecutive C_dyn_ measurements from each case was calculated and used for analysis. Descriptive statistics were also performed to summarize the data. The distribution of C_dyn_ was visually evaluated and tested for normality using both the Shapiro-Wilk and the Anderson-Darling normality tests.

In response to the substantial variance observed, and to comprehensively analyse the impact of the various factors on C_dyn_, a multiple linear regression model was developed.

First, a simple linear model was created using the “lm” function from the “lme4” package to analyse the relationship between C_dyn_ (dependent variable) and body mass (predictor variable). The relationship between C_dyn_ and body mass was graphically represented with the linear regression line and 95% confidence interval. For comparison, previously published linear regressions lines were also reported (12, 13).

Further analysis was conducted on variables considered likely to affect C_dyn_ in the order of anticipated importance, as determined by the authors. Initially, a correlation matrix was created to identify potential multicollinearities between the factors (e.g., BCS and morphology). Multicollinearities occur when two or more variables are highly correlated with each other. Specifically, if the absolute correlation coefficient between any two variables was >0.4, their relationship was investigated further and possible transformations or exclusions were considered.

Of the many factors assessed, a collinearity was expected between body mass and internal diameter of oro-tracheal tubes (18). Nevertheless, a marked effect of the internal diameter of the orotracheal tubes on C_dyn_ was suspected, therefore, it was considered relevant to keep this parameter after transformation. Because of multicollinearities, the deviation from the mean diameter of the investigated population rather than the absolute value of the diameter was evaluated. A graph plotting the internal diameter of the orotracheal tubes and the body mass was generated and three types of models were considered to model their relationship: root models, logarithmic models, and excitatory Emax models. Both difference and ratio of the internal diameter of the tube to its “predicted value” (i.e., the internal diameter expected from the various models for a given body mass) were investigated for each subject. The various approaches were compared using linear regression model metrics (AIC, Akaike Information Criterion; BIC, Bayesian Information Criterion; adjusted *R*^2^, Adjusted Coefficient of Determination) and ANOVA (Analysis of Variance) in R. A direct linear regression of the continuous variable or the use of cut-off values on the difference or ratio to create categories of internal diameters of orotracheal tubes (Low, Medium, and High) were tested and the best-fit was selected.

Multicollinearity was also expected between the length of the orotracheal tubes and their internal diameter, as well as with body mass. As, according to the Hagen-Poiseuille equation, the length of the orotracheal tubes should physically demonstrate less potential to influence C_dyn_ than their internal diameters, it was decided to test the length of the orotracheal tubes only if their internal diameter had no significant effect on the C_dyn_ adjusted to body mass model and to ignore it otherwise.

After addressing multicollinearities, the remaining individual variables were characterized through descriptive statistics and graphical representations (frequency histograms to describe the representativity of the eventual various subcategories within each variable). Direct relationship to C_dyn_ was evaluated using median boxplot, Wilcoxon rank-sum (if there were only two categories within the factors considered) or Kruskal-Wallis test followed by *post-hoc* Dunn's test if necessary for pairwise comparisons (for factors with more than two categories) and/or linear regression. Transformation of the data to create simplified categories was tested where appropriate and the final variable selected based on best model metrics (see below). The significance of each variable's addition to the linear regression of C_dyn_ adjusted to body mass was tested to identify potential candidates for inclusion in the multiple linear regression model. During this first selection of the variables, violation of conditions for linear regression were not tested.

Finally, a multiple linear regression model was built using a stepwise forward selection and backward elimination technique. Cases containing missing or obviously incorrect information in one of the categories were excluded from the analysis for that category only, and if the variable was included in the final model. Decision on candidates' selection was made based on the overall picture. The predictors were removed and added one by one according to anticipated relevance based on their variable-specific *p*-values. At each step, the AIC, BIC, adjusted *R*^2^, and *p*-values (ANOVA model comparison) were evaluated. For AIC and BIC, lower values were preferred for better model fit. For adjusted *R*^2^, higher values indicated a better fit. Linearity, homoscedasticity, independence of residuals, normality of residuals and multicollinearity were assessed using residuals plots, the Durbin-Watson statistic, the Shapiro-Wilk test, and variance inflation factors, respectively.

Statistical significance was considered when *p* < 0.05.

## 3 Results

A total of 515 cases were recruited. Data from 12 cases in which C_dyn_ varied by more than 15% across the three measurements were excluded from the analysis. Cases with missing or obviously incorrect data in selected variables were excluded when analyzing variables independently, and from the total dataset only after their inclusion in the final model was confirmed. This approach resulted in varying case numbers across analyses, as detailed in relevant sections of the Supplementary data. A total of 462 cases were included in the final model.

A variety of breeds was represented. Table 1 lists the breeds and the number of dogs of each breed included in the study. Due to insufficient numbers of each breed, the effect of breed on C_dyn_ was not investigated.

**Table 1:** Breeds and number of individuals.

**Breed**	**Number**
Mixed breed	95
French Bulldog	37
Labrador Retriever	36
English Springer Spaniel	18
Cocker Spaniel	16
Golden Retriever	14
Dachshund	13
German Shepherd, Pug	12 each
Beagle, Border Collie, Boxer, Staffordshire Bull Terrier	10 each
Chihuahua	9
Jack Russell Terrier, Shih Tzu	8 each
Dalmatian, English Bulldog, Whippet	7 each
Boston Terrier	6
American Staffordshire Terrier, Bernese Mountain Dog, Lurcher, Maltese, Yorkshire Terrier	5 each
Border Terrier, Rhodesian Ridgeback	4 each
Australian Shepherd, Bichon Frisé, Bolonka Zwetna, Cavalier King Charles Spaniel, Cockerpoo, Doberman Pinscher, Greyhound, Labradoodle, Lhasa Apso, Miniature Dachshund, Newfoundland, Rottweiler, Siberian Husky, West Highland White Terrier	3 each
American Bulldog, Beauceron, Cairn Terrier, Cane Corso, Cavapoo, Chow-Chow, Collie, Dogue de Bordeaux, Flat Coated Retriever, Gascon Saintongeois, Great Dane, Lagotto Romagnolo, Miniature Schnauzer, Norfolk Terrier, Pembroke Welsh Corgi, Pomeranian, Saint Bernard, Schnauzer, White Swiss Shepherd	2 each
Akita Inu, Anatolian Shepherd dog, Australian Cobberdog, Basset Hound, Bearded Collie, Belgian Shepherd, Bergamasco Shepherd, Biewer Terrier, Dutch Shepherd, German Short Haired Pointer, German Spaniel, Gordon Setter, Greater Swiss Mountain Dog, Griffon, Hovawart, Irish Soft Coated Wheaten Terrier, Irish Terrier, Krömfohrländer, Leonberger, Malinois, Miniature Bull Terrier, Miniature Poodle, N/A, Old English Sheepdog, Old English Bulldog, Pitt bull terrier, Plummer Terrier, Pointer, Royal Puddle, Shetland Sheepdog, Shiba Inu, Standard Poodle, Tibetan Terrier, Toy Poodle, Weimaraner, Welsh Corgi Cardigan	1 each

Distribution of breeds and number of individuals for each breed in a total of 462 dogs that were retained in the final statistical analysis. The data was collected from 11 centers across six countries in a study aiming to establish the reference interval of dynamic compliance (C_dyn_) and identify variables that have the potential to influence C_dyn_.

Based on insufficient data, the following categories were not included in the analysis: Trendelenburg (only four animals were positioned in Trendelenburg and eight in inverted Trendelenburg); breathing system type (circle breathing systems were used in most dogs; the use of a Mapleson A and D were reported in only one and seven cases, respectively); the administration of etomidate, desflurane and intravenous lidocaine (reported in only eight, one and 19 cases, respectively).

Descriptive statistics and the frequency histogram of C_dyn_ are reported in Table 2 and as Supplementary material (Section 1 and Supplementary Figure 1). Normal distribution was rejected.

**Table 2:** Descriptive statistics of C_dyn_ (in mL/cmH_2_O) in a cohort of 515 dogs anesthetized in 11 centers across six countries.

**Dynamic compliance of the respiratory system in anesthetized dogs**			
*n* = 515			
**Mean**	**Standard deviation**		
29.83	19.9		
**Median**	**Minimum**		**Maximum**
24.67	2.33		106.33
**Range**	**Skew**	**Kurtosis**	**Standard error**
104	1	0.68	0.8

Anesthetic management was at the discretion of the local anesthesia team, and the dogs were undergoing clinical procedures based on their individual conditions.

**Figure 2:**
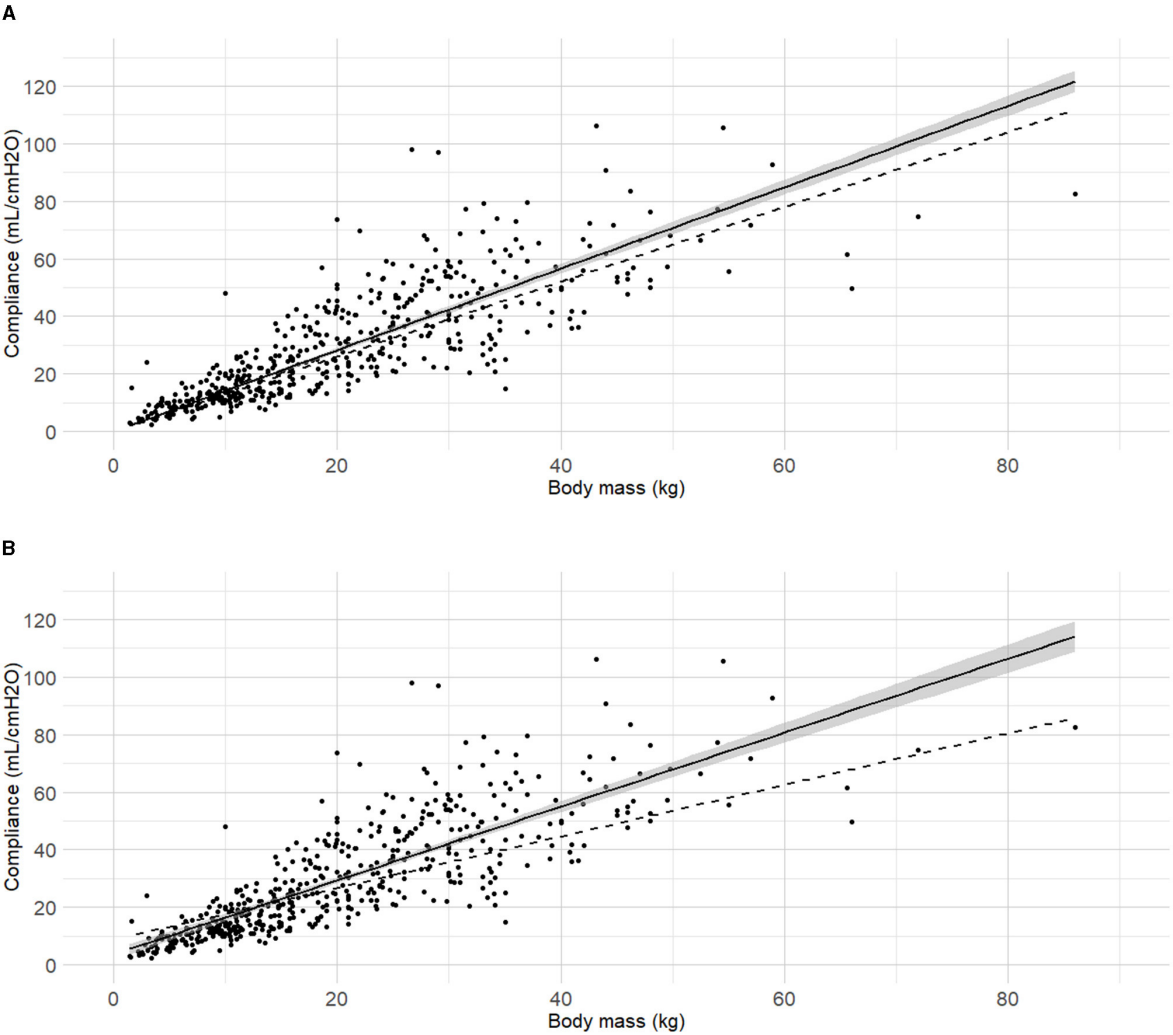
**Best fitting linear regressions dynamic compliance (C**_**dyn**_**) against body mass. (A)** Best-fitting linear regression of C_dyn_ against body mass crossing the origin (continuous line, 95% confidence interval) in a cohort of 515 dogs anaesthetized in 11 centers across six countries. Anaesthetic management was at the discretion of the local anaesthesia team, and the dogs were undergoing clinical procedures based on their individual conditions. The model from Asorey et al. (12) is reported in the figure (dashed line) for comparison. **(B)** Best-fitting linear regression of C_dyn_ against body mass with free intercept (continuous line, 95% confidence interval), and the model from Bradbrook et al. (13) (dashed lines) for comparison.

Body mass was selected as the first predictor variable for C_dyn_ based on prior research indicating a significant relationship between these variables. The line of best fit of C_dyn_ against body mass crossing the origin and the model from Asorey et al. (12) for comparison are presented in Figure 2A. The line of best fit of C_dyn_ against body mass with free intercept and the model published by Bradbrook et al. (13) for comparison are presented in Figure 2B. Both linear models and their comparison are reported as Supplementary material (Section 2 and Supplementary Tables 1A, B). The model with intercept was kept for further development according to better performance metrics (Supplementary Table 1C).

**Figure 3:**
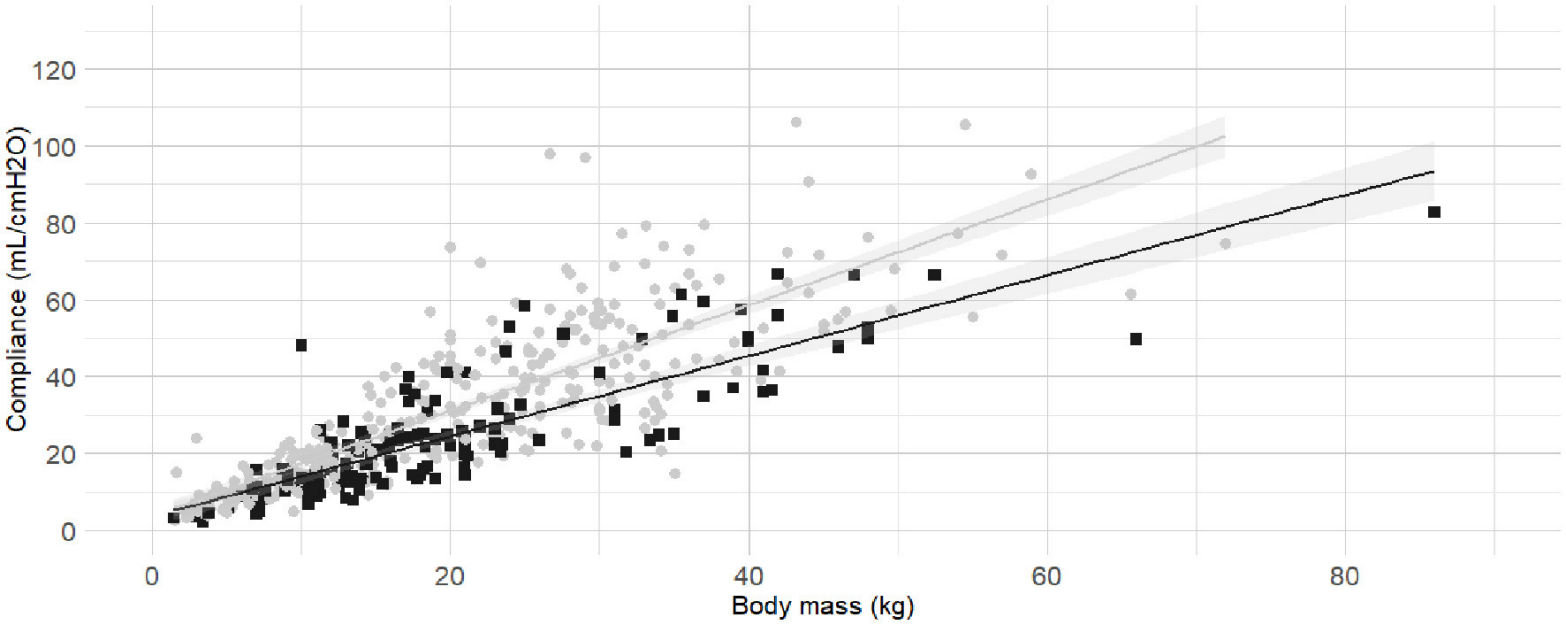
**Linear regressions of dynamic compliance (C**_**dyn**_**) against body mass for the subgroups with or without small orotracheal tubes**. Best-fitting linear regressions of C_dyn_ against body mass for the subgroup of dogs having “small” endotracheal tubes (internal diameters of the tubes below 7% smaller than the predicted value, black squares and line) or “appropriate and large” endotracheal tubes with internal diameters ±6% of predicted value and >7% of predicted value (gray circles and line) in a cohort of 494 dogs anaesthetized in 11 centers across six countries. Anaesthetic management was at the discretion of the local anaesthesia team, and the dogs were undergoing clinical procedures based on their individual conditions.

The internal diameter of the orotracheal tubes was the second factor in the order of anticipated importance determined by the authors. As the correlation coefficient between body mass and internal diameter of the orotracheal tubes was 0.4, further investigation was warranted before the relationship between internal diameter of the orotracheal tubes and C_dyn_ was analyzed. Detailed results for the relationship between internal diameter of the orotracheal tubes and body mass are presented as Supplementary material (Section 3, Supplementary Table 2 and Supplementary Figure 2). The best fitting prediction of orotracheal tube against body mass was a sigmoid Emax model (Supplementary Section 4 and Supplementary Figure 3). Based on best models' performance metrics, internal diameter was categorized as “Small” if more than 7% smaller than its predicted value, or “Medium/large” if above. The Figure 3 represents the C_dyn_ against the body mass with the linear regressions. The effect of the internal diameter of the orotracheal tube on the C_dyn_ ~ body mass model is reported in Table 3.

**Table 3:** Linear regression models for dynamic compliance (C_dyn_) in anesthetized dogs.

**Best fitting linear regression**	**Adjusted *R*^2^**	**AIC**	**BIC**	***p*-value**	**Graphical representation**
C_dyn_ ~ Mass	0.659	3,898.02	3,910.68		Figure 2B
C_dyn_ ~ Mass, ETT	0.679	3,797.00	3,813.00	<0.001	Figure 3
C_dyn_ ~ Mass, BCS^*^	0.679	3,774.07	3,795.05	<0.001	Figure 4
C_dyn_ ~ Mass, Morphology	0.669	3,812.39	3,833.41	<0.001	Figure 5
C_dyn_ ~ Mass, Position^**^	0.658	3,813.35	3,830.14	0.036	Figure 6
C_dyn_ ~ Mass, Sensor	0.666	3,795.49	3,816.47	0.001	Figure 7
C_dyn_ ~ Mass, FIO_2_>80%_10 minutes	0.661	3,807.74	3,824.54	0.005	Figure 8
C_dyn_ ~ Mass, Time post induction	0.664	3,740.64	3,757.37	<0.001	Figure 9
C_dyn_ ~ Mass, Inspiratory time	0.676	3,606.53	3,623.14	<0.001	Figure 10
C_dyn_ ~ Mass, Ventilation mode	0.659	3,790.22	3,806.99	0.011	Figure 11

Best-fitting linear regression models for C_dyn_ ~ Body mass and subsequent models incorporating additional factors to improve fit (orotracheal tube smaller than 7% of the predicted internal diameter for that body mass, body condition score, morphology, the lateral position, the sensor used to determine C_dyn_, the administration of >80% oxygen for at least 10 minutes before the measurements, the inspiratory time and the respiratory mode. Metrics reported include Akaike Information Criterion (AIC), Bayesian Information Criterion (BIC), adjusted R^2^, and p-values, along with the figure number. Data from a cohort of 515 dogs under general anesthesia, collected from 11 centers across six countries, were analyzed to establish the reference interval of dynamic compliance (C_dyn_) and identify potential influencing variables.

^*^Three categories: low, medium, and high BCS.

^**^Two categories: lateral or not.

**Figure 4:**
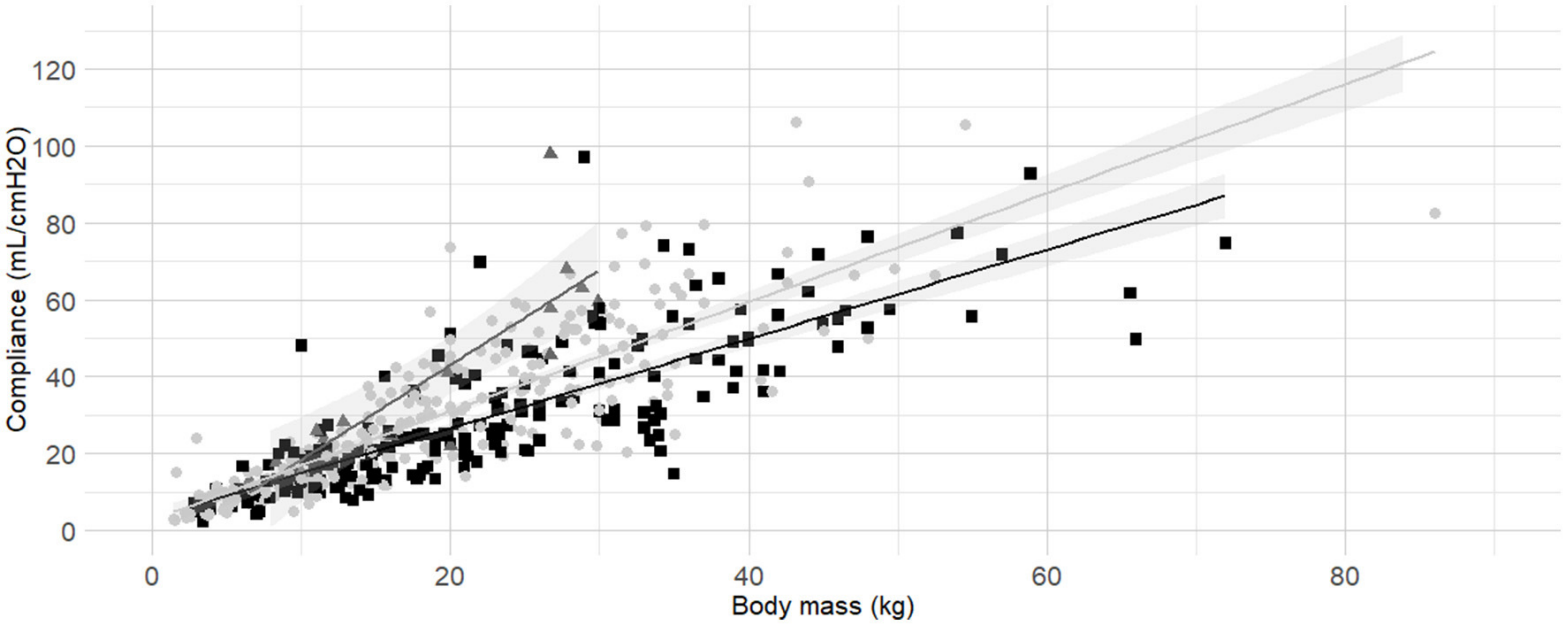
**Linear regressions of dynamic compliance (C**_**dyn**_**) against body mass for the subgroups low, medium and high Body Condition Score (BCS)**. Best-fitting linear regressions of C_dyn_ against body mass for the for the subgroup of low (dark gray triangles and line, BCS 1–3), medium (light gray circles and line, BCS 4–5), and high BCS (black squares and line, BCS 6-9) in a cohort of 491 dogs anaesthetized in 11 centers across six countries. Anaesthetic management was at the discretion of the local anaesthesia team, and the dogs were undergoing clinical procedures based on their individual conditions.

**Figure 5:**
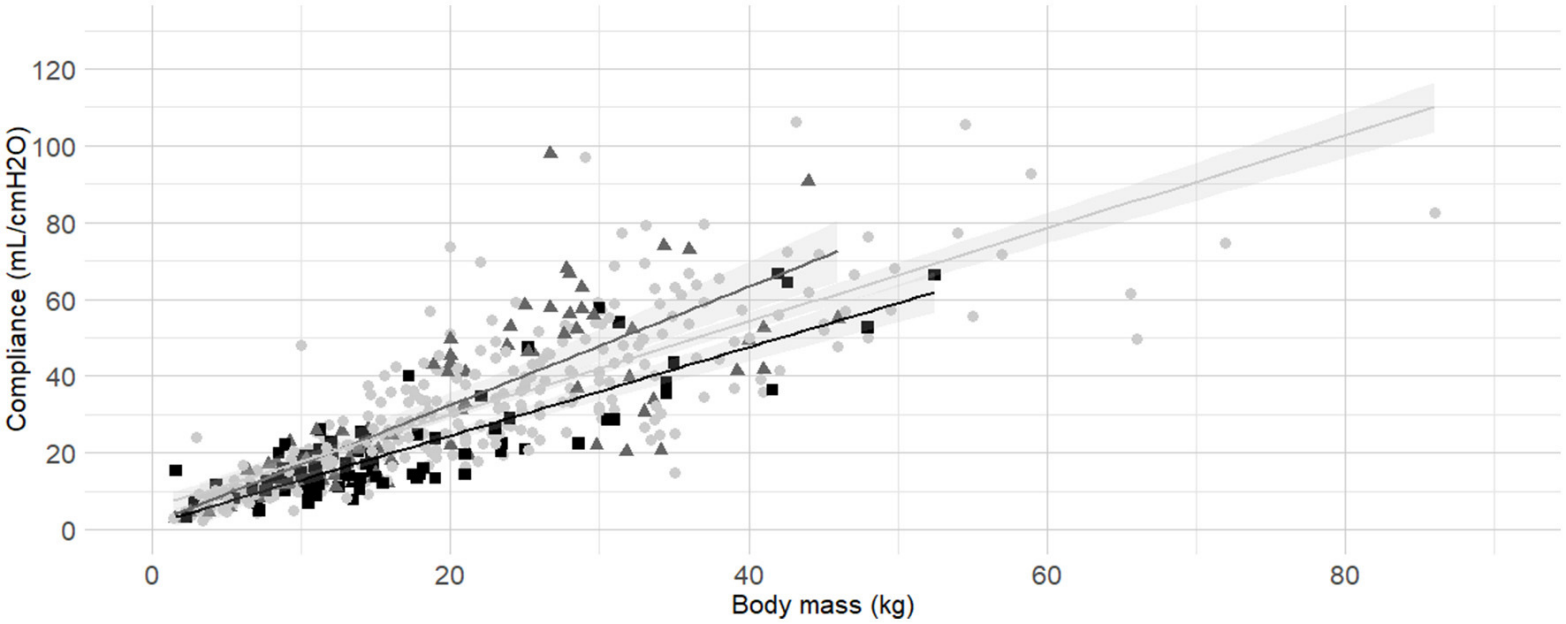
**Linear regressions of dynamic compliance (C**_**dyn**_**) against body mass for the subgroups brachymorphic, dolichomorphic and mesomorphic**. Best-fitting linear regressions of C_dyn_ against body mass for the for the subgroup of of morphology including dolichomorphic (dark gray triangles and line), mesomorphic (light gray circles and line), and brachymorphic (black squares and line) in a cohort of 494 dogs anaesthetized in 11 centers across six countries. Anaesthetic management was at the discretion of the local anaesthesia team, and the dogs were undergoing clinical procedures based on their individual conditions.

**Figure 6:**
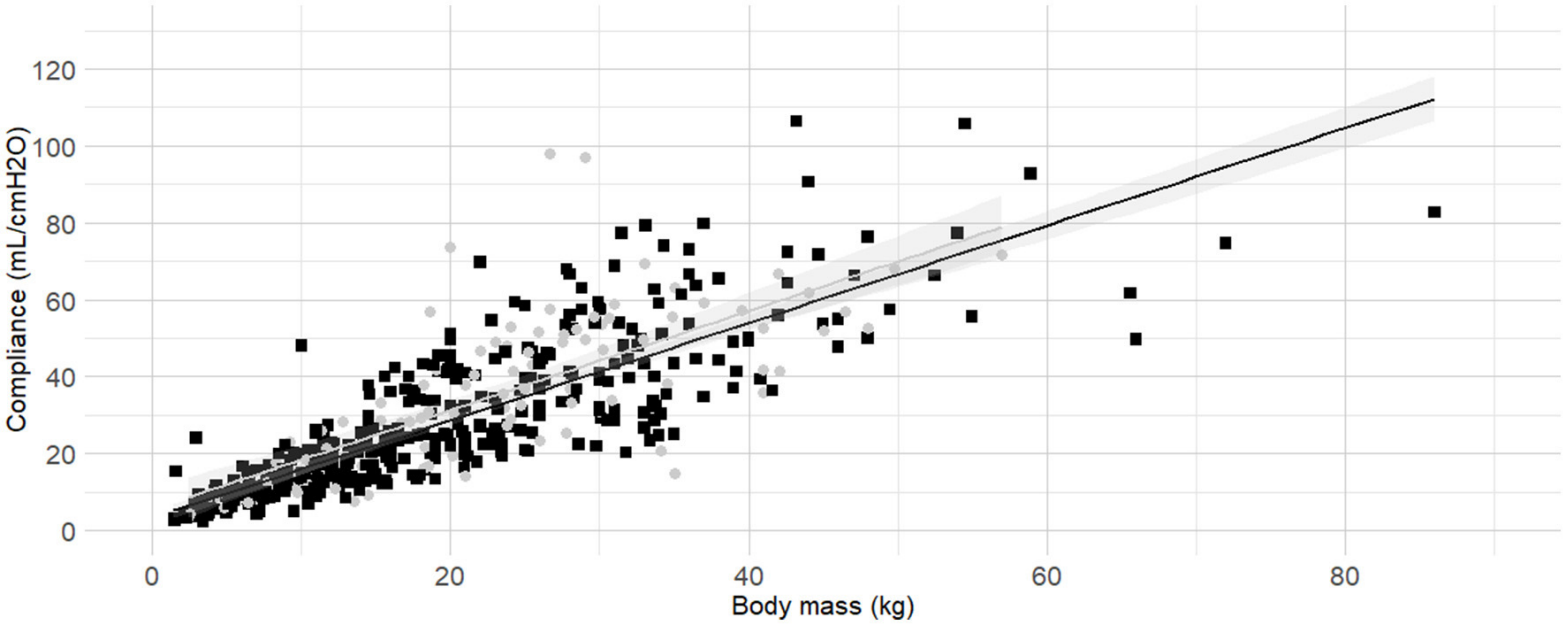
**Linear regressions of dynamic compliance (C**_**dyn**_**) against body mass in lateral recumbency or not**. Best-fitting linear regressions of C_dyn_ against body mass in lateral recumbency (gray circles and line) or in another position (black squares and line) in a cohort of 492 dogs anaesthetized in 11 centers across six countries. Anaesthetic management was at the discretion of the local anaesthesia team, and the dogs were undergoing clinical procedures based on their individual conditions.

**Figure 7:**
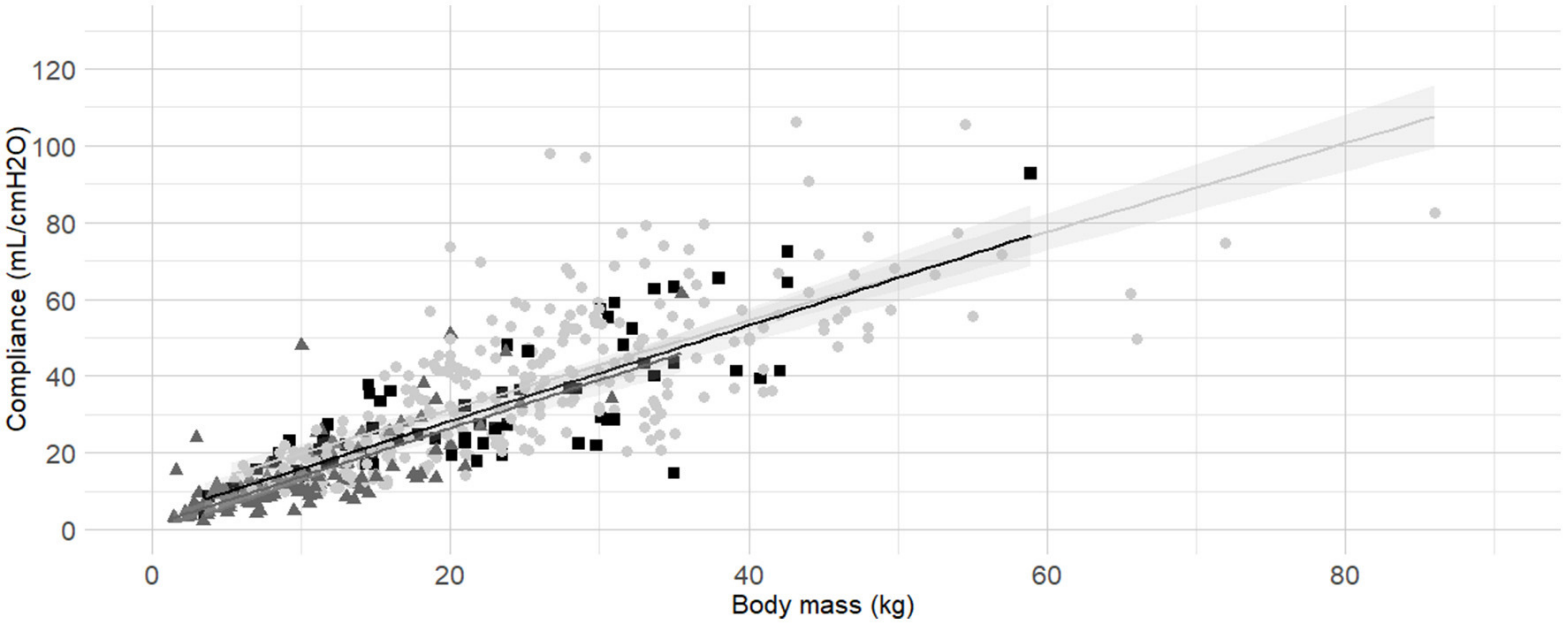
**Linear regressions of dynamic compliance (C**_**dyn**_**) against body mass according to the sensor used to establish C**_**dyn**_ **(Pedi-lite, D-lite or any other spirometry sensor)**. Best-fitting linear regressions of C_dyn_ against body mass according to sensors used to measure: D-Lite (light gray circles and line), Pedi-Lite (dark gray triangles and line), or others (black squares and line) in a cohort of 491 dogs anaesthetized in 11 centers across six countries. Anaesthetic management was at the discretion of the local anaesthesia team, and the dogs were undergoing clinical procedures based on their individual conditions.

**Figure 8:**
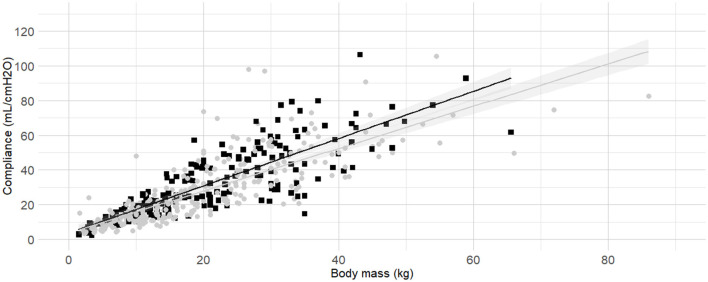
**Linear regressions of dynamic compliance (C**_**dyn**_**) against body mass according to the administration or not of an inspired fraction of oxygen (FIO**_**2**_**) above 80% for a minimum of 10 minutes before the measurements**. Best-fitting linear regressions of C_dyn_ against body mass according to the administration (grey circles and line) or not (black squares and line) of a FIO_2_ > 80% for at least 10 minutes before the measurement in a cohort of 492 dogs anaesthetized in 11 centers across six countries. Anaesthetic management was at the discretion of the local anaesthesia team, and the dogs were undergoing clinical procedures based on their individual conditions.

**Figure 9:**
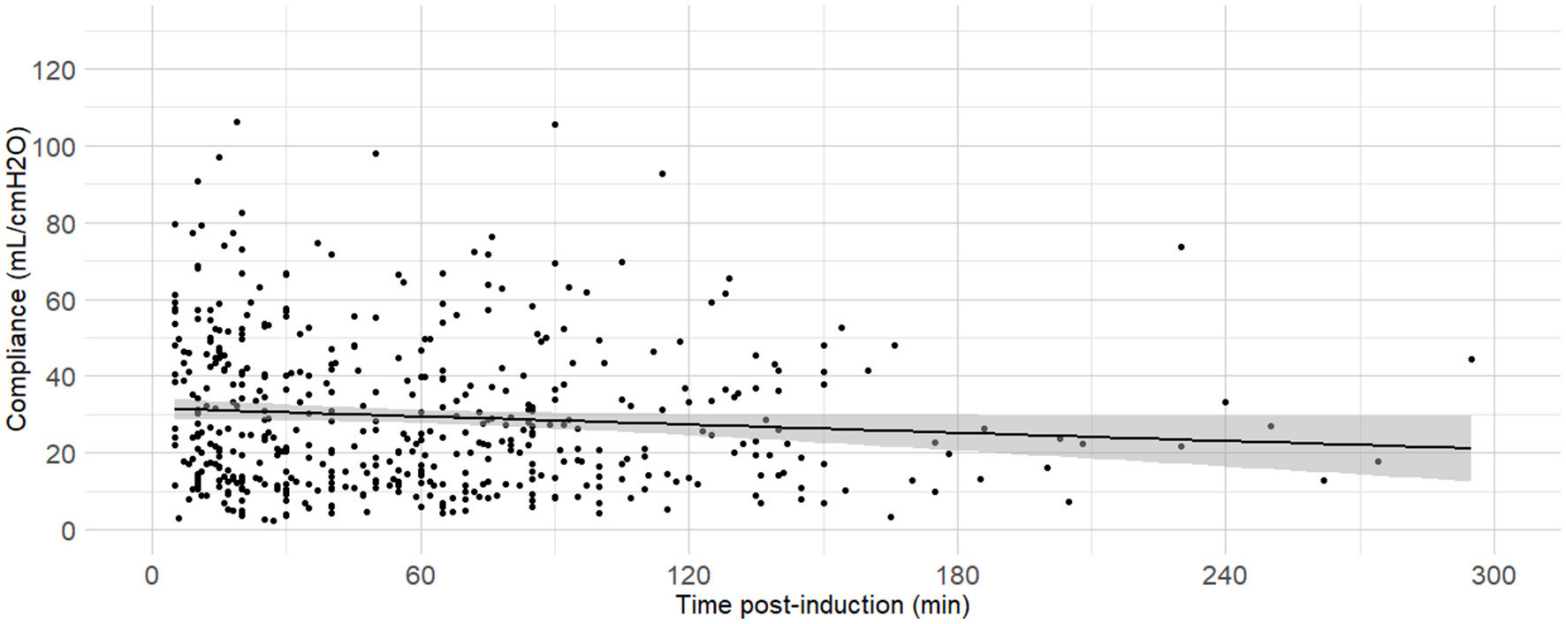
**Linear regressions of dynamic compliance (C**_**dyn**_**) against the time between anaesthesia induction and the measurements**. Best-fitting linear regressions of C_dyn_ against time (in minutes) between induction of general anaesthesia and the C_dyn_ determination in 484 dogs anaesthetized in 11 centers across six countries. Anaesthetic management was at the discretion of the local anaesthesia team, and the dogs were undergoing clinical procedures based on their individual conditions.

**Figure 10:**
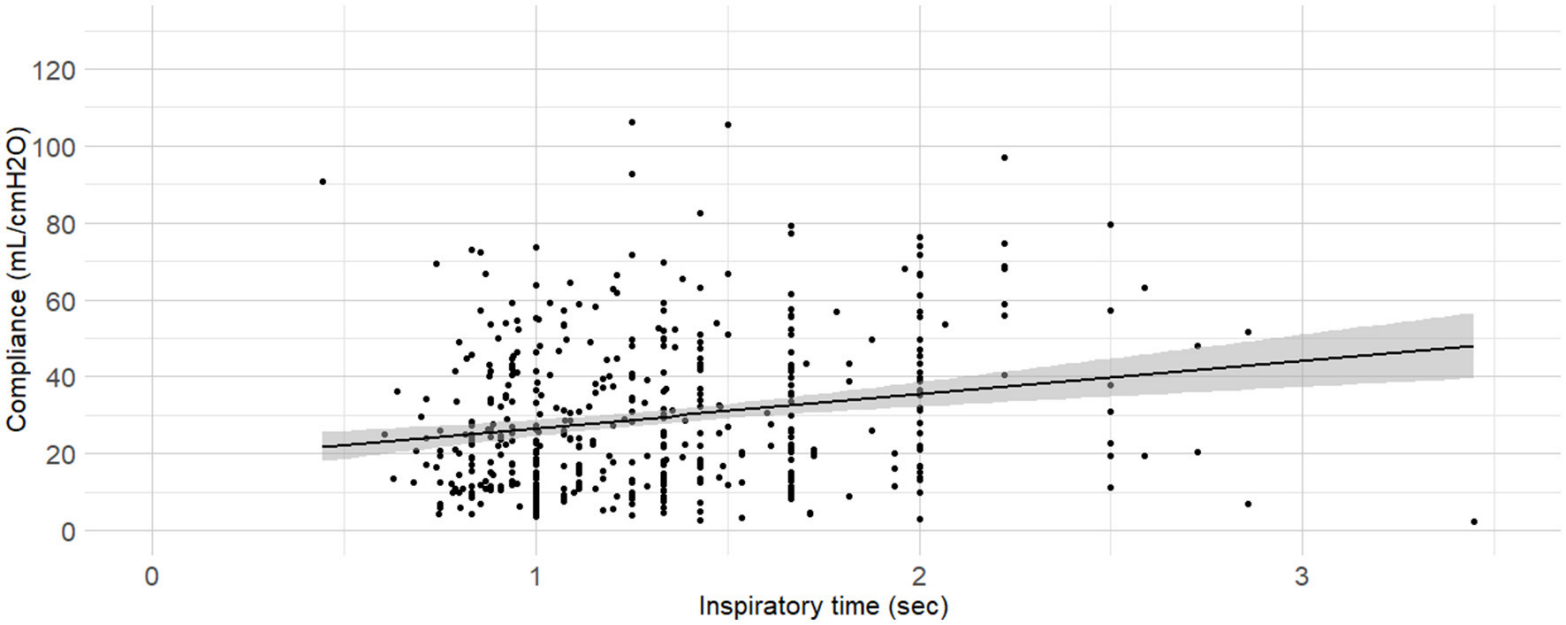
**Linear regressions of dynamic compliance (C**_**dyn**_**) against the inspiratory time set**. Best-fitting linear regressions of C_dyn_ against inspiratory time set on the ventilator (in minutes) at the time of C_dyn_ determination in 470 dogs anaesthetized in 11 centers across six countries. Anaesthetic management was at the discretion of the local anaesthesia team, and the dogs were undergoing clinical procedures based on their individual conditions.

**Figure 11:**
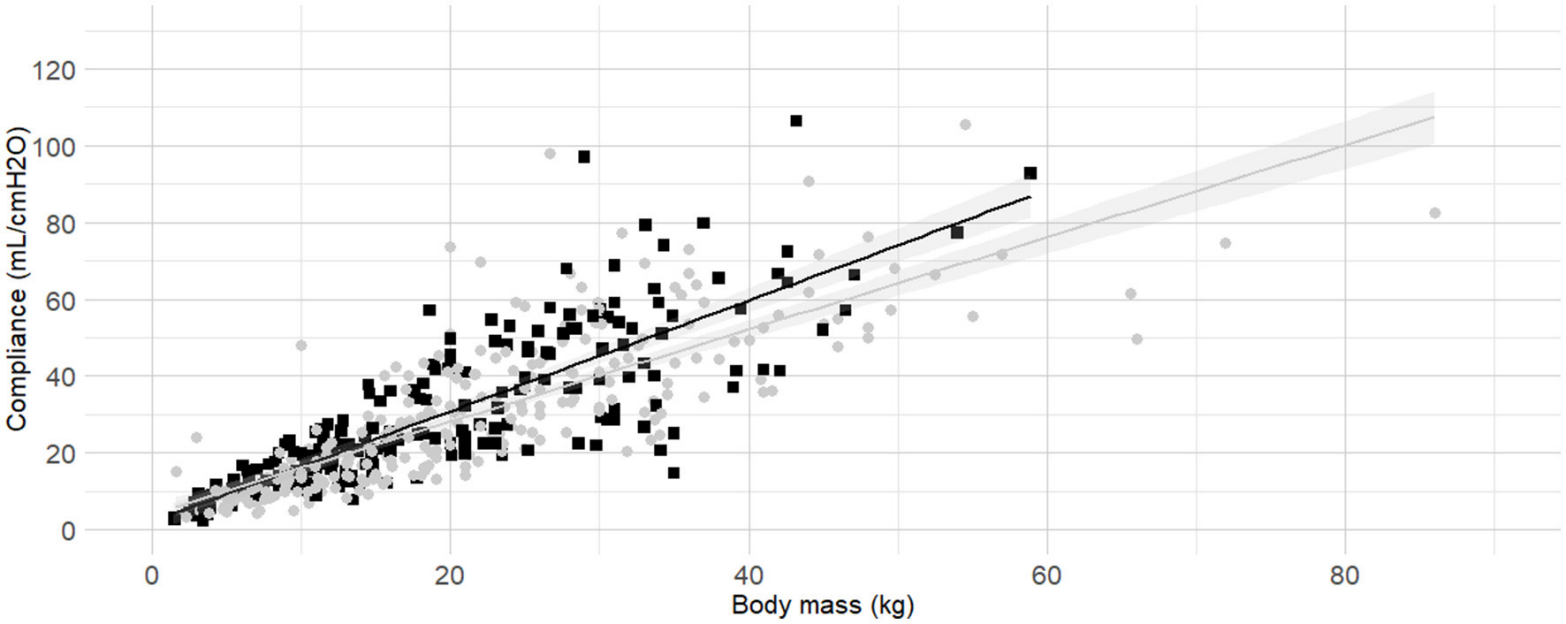
**Linear regressions of dynamic compliance (C**_**dyn**_**) against body mass according to the administration or not of an inspired fraction of oxygen (FIO**_**2**_**) above 80% for a minimum of 10 minutes before the measurements**. Best-fitting linear regressions of C_dyn_ against body mass according to the modes of ventilation set, volume-controlled ventilation (gray circles and line), pressure-controlled ventilation (black squares and line) in a cohort of 489 dogs anaesthetized in 11 centers across six countries. Anaesthetic management was at the discretion of the local anaesthesia team, and the dogs were undergoing clinical procedures based on their individual conditions.

The correlation coefficients between the length of the orotracheal tubes and their diameters and the length of the orotracheal tubes and body mass were 0.46 and 0.74, respectively. As the internal diameter of the orotracheal tubes impacted the C_dyn_ ~ body mass model, the influence of the length of the orotracheal tube was not investigated. No other multicollinearity was identified (i.e., the absolute correlation coefficient between all the other variables were <0.4).

Body condition score was also identified as factor influencing C_dyn_. According to model comparisons, the BCS were best divided into the following three categories: “low BCS” (1/9, 2/9 and 3/9), “medium BCS” (4/9 and 5/9), and “high BCS” (6/9, 7/9, 8/9 and 9/9). The frequency histogram of BCS, boxplots of C_dyn_ for each BCS are presented in Supplementary material (Section 6, Supplementary Figures 5A–C). Linear regressions of C_dyn_ against body mass for the subgroup of “low BCS,” “medium BCS,” and “high BCS” were calculated (Table 3) and graphically represented (Figure 4).

The following variables had a significant impact on C_dyn_ and improved the C_dyn_ ~ body mass model when considered individually (i.e., reduction in both AIC and BIC, and *p* < 0.05): morphology, lateral recumbency, spirometry sensor used, the administration of FIO_2_ >80% for at least 10 minutes before the measurements, time elapsed between anesthesia induction and the measurements, inspiratory time set on the ventilator, and ventilation mode. Their effect on the C_dyn_ ~ body mass model and associated figures is reported in Table 3. Additional information on the individual variables can be found in the Supplementary material (Section 7 and Supplementary Figures 6A, B; Section 8 and Supplementary Figures 7A, B; Section 9 and Supplementary Figures 8A, B; Section 12 and Supplementary Figures 11A, B; Section 13 and Supplementary Figure 12; Section 14 and Supplementary Figure 13; Section 15 and Supplementary Figures 14A, B).

The development of the multiple linear regression model involved using stepwise forward selection and backward elimination techniques. The procedures and criteria for model selection are detailed in Supplementary material (Section 17 and associated Supplementary Tables). Variables were added or removed iteratively, with the goal of achieving a model that balanced fit and simplicity. The final model is presented in Table 4. The variables included were selected based on their contribution to the model's performance, as reflected in the evaluation criteria described. The best-fitting model identified a linear relationship between C_dyn_ and body mass when the following conditions were met: a “high BCS,” “small” orotracheal tubes (<7% smaller than predicted), the use of a D-lite flow sensor, and the absence of a high FIO_2_ (>80%) exposure for more than 10 minutes prior to C_dyn_ measurement. In cases where these conditions were not met, additional factors needed to be incorporated into the model. Factors such as a “Low (1/9, 2/9, 3/9) and Medium (4/9, 5/9) BCS,” an orotracheal tube with an internal diameter not <7% smaller than the predicted value (i.e., an orotracheal tube of the predicted size or larger) and longer inspiratory times were associated with increased C_dyn_. Conversely, the use of alternative spirometry sensors, including Ped-lite, or prolonged exposure to high FIO_2_ levels resulted in decreased C_dyn_.

**Table 4:** Linear regression models for dynamic compliance (C_dyn_) in anesthetized dogs.

**Variable**	**Initial estimate**	**Initial SE**	**Initial *p*-value**	**Corrected mean estimate**	**Estimate 95%CI**	**Robust SE**	**Corrected *p*-value**
Intercept	−3.078	2.416	0.203	−3.011	[−7.962, 1.539]	2.511	0.221
Body mass	+1.166	0.046	<0.001	+1.164	[1.049, 1.298]	0.066	<0.001
ETT_ID (medium)	+5.971	1.088	<0.001	+6.015	[4.010, 7.837]	0.994	<0.001
BCS (Low)	+9.293	3.010	0.002	+9.251	[5.157, 13.834]	2.288	<0.001
BCS (Medium)	+3.705	1.002	<0.001	+3.708	[1.707, 5.740]	1.035	<0.001
Inspiratory time (in seconds)	+4.708	1.148	<0.001	+4.681	[2.081, 7.572]	1.436	0.001
Sensor (Other)	−4.506	1.650	0.007	−4.538	[−7.676, −1.222]	1.677	0.007
Sensor (Pedi–Lite)	−4.992	1.336	<0.001	−5.028	[−7.129, −2.799]	1.142	<0.001
FIO_2_ >80% for at least 10 minutes (Yes)	−2.933	1.186	0.014	−2.951	[−5.259, −0.548]	1.206	0.015

Final model for C_dyn_ per body mass, derived using stepwise forward selection and backward elimination techniques. The models incorporate additional factors to improve fit, including orotracheal tube smaller than 7% of the predicted internal diameter for that body mass, body condition score, morphology, lateral position, sensor used to determine C_dyn_, administration of >80% oxygen for at least 10 minutes before the measurements, inspiratory time, and respiratory mode. Data were analyzed from a cohort of 462 dogs under general anesthesia, collected from 11 centers across six countries. Anesthetic management was at the discretion of the local anesthesia team, and the dogs were undergoing clinical procedures based on their individual conditions. Corrected mean estimates and their confidence intervals (95% CI) were obtained from bootstrapping (n = 10,000). Robust standard errors (SE) and corrected p-values were obtained using Davidson and Mackinnon heteroscedasticity-consistent estimators.

**Figure 12:**
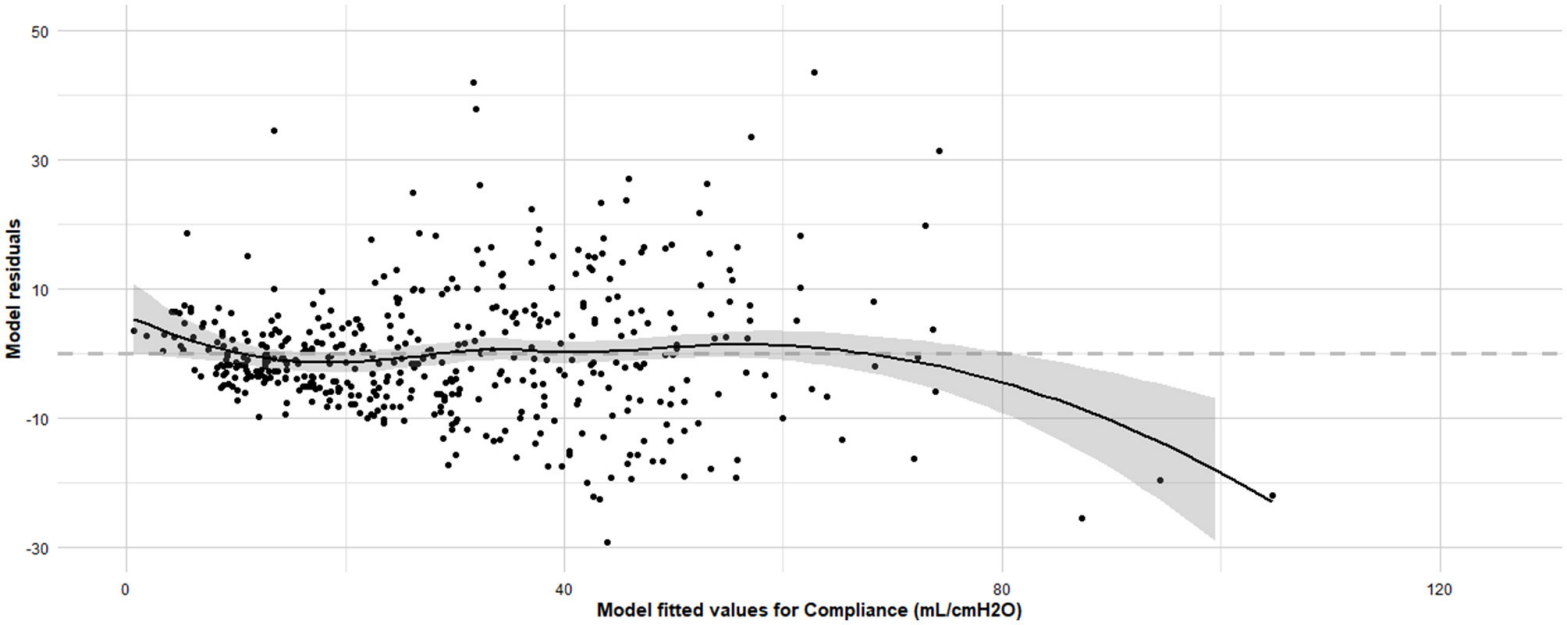
**Residuals vs. fitted values plot for the final linear regression model assessing dynamic compliance (C**_**dyn**_**) in anaesthetized dogs**. Residuals vs. fitted values plot for the final linear regression model assessing C_dyn_ in anaesthetized dogs and the Loess smoothing trend curve (black solid line) and its 95% confidence bands (gray shadow). The x-axis represents the fitted values obtained from the regression model, and the y-axis represents the residuals, which are the differences between the observed and predicted C_dyn_ values. The grey horizontal line at zero aids in visualizing the spread and pattern of residuals. Ideally, residuals should be randomly scattered around this line, indicating that the assumptions of linearity, homoscedasticity, and independence are met. The model was developed using stepwise forward selection and backward elimination, incorporating factors such as orotracheal tube size, body condition score, morphology, lateral position, sensor type, high oxygen administration for 10 minutes before the measurements, inspiratory time, and respiratory mode. The data analyzed were from a cohort of 462 dogs under general anaesthesia, collected from 11 centers across six countries.

**Figure 13:**
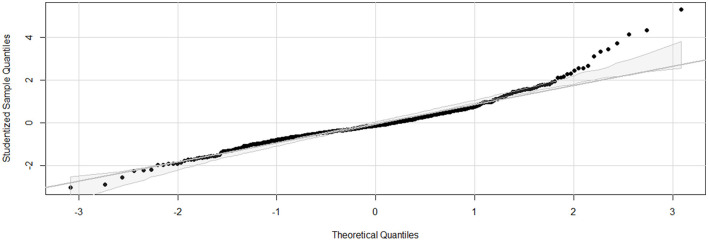
**Q-Q (Quantile-Quantile) plot for the final linear regression model assessing dynamic compliance (C**_**dyn**_**) in anaesthetized dogs**. Q-Q plot for the final linear regression model assessing C_dyn_ in anaesthetized dogs. The x-axis represents the theoretical quantiles from a normal distribution, and the y-axis represents the studentized residuals from the regression model. The diagonal line represents the expected quantiles if the residuals follow a normal distribution. Points that deviate from this line indicate departures from normality. The model was developed using stepwise forward selection and backward elimination, incorporating factors such as orotracheal tube size, body condition score, morphology, lateral position, sensor type, high oxygen administration for 10 minutes before the measurements, inspiratory time, and respiratory mode. The data analyzed were from a cohort of 462 dogs under general anaesthesia, collected from 11 centers across six countries.

Linearity was confirmed (Residuals Plot, Figure 12). Homoscedasticity was not confirmed (*p* < 0.001). Independence was confirmed (Durbin-Watson test, *p* = 0.486). Normality of Residuals (QQ Plot, Figure 13) was not confirmed (Shapiro-Wilk test, *p* < 0.001). Multicollinearity was confirmed (Variance inflation factors < 1.3). To account for heteroscedasticity, robust standard errors were calculated using the more conservative Davidson and Mackinnon heteroscedasticity-consistent estimators (via the *Sandwich* package in R). The results, including recalculated *p*-values, are presented in Table 4. Additionally, to address the non-normality of the residuals, estimates were re-evaluated through bootstrapping (*n* = 10,000; *Boot* package in R) to obtain bias-corrected estimates and 95% confidence intervals (Table 4).

Age, the presence or absence of HMEs, the F_I_O_2_ and the PEEP levels did not have any significant effect when integrated to the multiple regression linear model. Additional information on the individual variables can be found in the Supplementary material (Section 5 and Supplementary Figures 4A, B; Section 10 and Supplemental Figures 9A–C; Section 11 and Supplemental Figures 10A, B; Section 16 and Supplemental Figures 15A, B). The administration of alfaxalone, alpha-2 adrenergic receptor agonists, anti-muscarinic, isoflurane, propofol, and sevoflurane had a significant effect on C_dyn_ (Yes/No for each drug; Wilcoxon rank-sum test *p*-value = 0.012, 0.012, 0.002, 0.007, 0.013, and 0.036, respectively). However, their individual effect was minimal to negligible on the C_dyn_ ~ body mass model and they were not included in the final model. The ventilation mode (Supplementary Section 15 and Supplementary Figures 14A, B), the administration of acepromazine, benzodiazepines, butorphanol, ketamine, mu-agonist opioids and neuromuscular blocking agents and the use of loco-regional blocks or epidural anesthesia did not significantly influence C_dyn_ (Yes/No for each drug or technique; Wilcoxon rank-sum test *p*-value = 0.711, 0.841, 0.222, 0.368, 0.811, 0.442, 0.235, 0.861, and 0.735, respectively).

## 4 Discussion

The main aim of this study was to establish a reference interval for C_dyn_ in anesthetized dogs. The large variance detected in C_dyn_ indicated that it was too inaccurate to develop a reference range and that modeling was required to estimate C_dyn_. Our findings demonstrated that C_dyn_ could be quantified relative to body mass. However, it was also notably affected by the internal diameter of orotracheal tubes, BCS, inspiratory time settings, the type of sensor used for C_dyn_ determination, and exposure to FIO_2_ >80% for at least 10 minutes prior to measurement. These findings illustrate that a single reference interval would be misleading or unhelpful in clinical practice due to the varying combinations of factors that can alter the measurement in different ways. Despite this, our study provides valuable insights into the factors affecting C_dyn_ in anesthetized dogs and emphasizes the need for careful consideration of these variables in both clinical and research settings.

Our results demonstrated a linear relationship between C_dyn_ and body mass in dogs, corroborating previously documented findings (11–13). This relationship was incorporated into all subsequent modeling. However, our study offered significant insights not captured in previous investigations and highlighted that body mass alone was insufficient to reliably predict C_dyn_. Our unique approach, derived from consultations with the veterinary anesthesia community (1), enabled us to further investigate prospectively the influence of numerous factors.

In our study, the impact of the inspiratory time on C_dyn_ was evaluated. We observed that varying inspiratory times significantly influenced the readings obtained. The characteristics of the inspiration have been shown to influence compliance values. Both C_stat_ and quasi-static compliance require an inspiratory pause. Varying inspiratory pauses of <3 s gave different values of quasi-static compliance and did not provide accurate C_stat_ values in healthy dogs (8). Here, the significant impact of the inspiratory time on C_dyn_ was demonstrated, confirming earlier findings (14). The first publication about D-lite flow sensors states that “some systematic error in compliance measurement is introduced if a ventilation mode without a pause and well-defined plateau pressure is used. However, even then, trends will be quite informative.” (19). In clinical anesthesia and in many studies, the inspiratory-to-expiratory ratio is set, and the respiratory rate is adjusted to maintain a target partial pressure of expired carbon dioxide. Clinicians wishing to monitor changes in C_dyn_ or quasi-static compliance over time should consider the importance of inspiratory time and keep it consistent in their assessments. The characteristics of the inspiratory settings (inspiratory time or the inspiratory-to-expiratory ratio and the respiratory rate set, and inspiratory pause) should also be systematically reported in research.

Our results also showed an influence of the internal diameter of orotracheal tubes on C_dyn_, as documented previously (13). However, Bradbrook et al. stated that “since ETT (Endotracheal Tube) diameter was probably dependent upon body size and therefore body weight, only body weight was investigated further by general additive modeling” (13). By including more dogs in our study, we were able to further investigate and model this relationship. This allowed us to identify a diameter cut-off value of 7% below the predicted value, below which C_dyn_ is negatively impacted. It is worth noting that monitors using multiple linear regression of the whole respiratory cycle to calculate or estimate C_dyn_ might be less affected by airway resistance (20). Further investigation is necessary to validate these findings across a wider range of monitoring technologies and techniques.

Interestingly, in our study an FIO_2_ above 80% for at least 10 minutes resulted in lower calculated values of C_dyn_. Previous research showed that administering a low FIO_2_ (40%) to ventilated dogs was associated with less atelectasis formation compared to administering a high FIO_2_ (21). Mathematical modeling of absorption atelectasis kinetics indicated that the process developed fairly rapidly, based on human data (22). The 10-minutes cut-off was chosen because a complete collapse would mathematically develop in <10 minutes if a 3-minutes pre-oxygenation was followed by 100% oxygen administration (22). The time spent at a high FIO_2_ was previously shown not to influence compliance (12). However, in that study, the dogs received a high FIO_2_ for 34 ± 18 minutes, with the duration of high FIO_2_ administration being the focus of the analysis. Our findings might reflect a clinical manifestation of absorption atelectasis, suggesting its rapid development in dogs *in vivo*, as supported by the mathematical model.

In our model, the ventilation mode did not influence C_dyn_. However, previous research described higher compliance values when pressure-controlled ventilation was used compared to volume-controlled ventilation (23). Lower peak airway pressures tend to develop with pressure-controlled ventilation compared to volume-controlled ventilation (24), presumably because decelerating flow patterns enhance ventilation distribution in lungs with varying mechanical properties (25). In their study, Fantoni et al. used C_stat_ with a 4-s inspiratory hold, reflecting improved lung compliance without the resistive component of the airway and orotracheal tube. They targeted similar tidal volumes (10 mL/kg) and PEEP (5 cmH_2_O) with both modes and measured airway pressure. This contrasts with how veterinary anesthetists typically set pressure-controlled ventilation, where pressure is most commonly targeted (1). The reasons for the observed differences are unclear, although variations in the settings of ventilation modes could be a contributing factor. Future studies employing more standardized ventilation strategies could further clarify this aspect. It is worth noting that the influence of inspiratory time on C_dyn_ has the potential to vary significantly between volume-controlled and pressure-controlled ventilation, which may contribute to explaining the discrepancies observed in our results.

Our study did not investigate thoracic shape and BCS in the same manner as Asorey et al. (12). The 20% inspiratory pause set in their study precludes direct comparison with our findings. However, they highlighted a reduction in compliance in overweight and “barrel-chested” dogs. Since our data collection began before their publication, we could not make a direct comparison of thoracic shapes. Although there is some overlap in the morphology categories (brachymorphic, dolichomorphic, and mesomorphic), direct comparison was not feasible. However, BCS remained in our model as in theirs. Our results indicated that the effect of morphology on C_dyn_ was not retained in the multivariable analysis, possibly because it did not provide additional relevance beyond the internal diameter of the orotracheal tubes. There was no correlation between morphology and the internal diameter of the orotracheal tubes used in our study, so this relationship was not investigated further.

This study has several limitations. Firstly, while a variety of spirometry technologies and monitors are commonly used in veterinary anesthesia (1), near-patient spirometers using Pedi-lite or D-lite flow sensors (Datex Ohmeda/GE Healthcare) were overrepresented. Additionally, several commonly available monitors were not used in this data collection. Unfortunately, no conclusions can be drawn regarding the impact of some monitors that were either underrepresented or not included in this study. Nevertheless, our model demonstrated an effect of technology used to determine C_dyn_ in this study and thus support the importance of considering the type of sensor when measuring compliance.

Secondly, while we acknowledge the potential for clustering due to the multicentre nature of the study, our primary aim was to assess dynamic compliance across a broad, representative sample rather than focus on center-specific effects. Incorporating a center variable would have required additional adjustment for the variability in machines used across centers, complicating the analysis without contributing significant clinical insight. Nonetheless, future studies could benefit from evaluating clustering effects, especially in contexts where center-based differences are of clinical relevance.

Thirdly, although calibration check of the monitors and correction of the data were originally planned, it proved practically impossible. It is likely that some of the monitors used in this study were not calibrated. However, this seems to represent typical veterinary anesthesia practice (1) thus the results similar expected findings in this environment.

Fourthly, although our survey among veterinary anesthetists documented the potential effect of some categories on C_dyn_ a few of them were not included in the analysis because they were not sufficiently represented (Trendelenburg; breathing system type; the administration of etomidate, desflurane, and intravenous lidocaine). Additionally, the limited number of cases in some categories may have caused certain variables, like PEEP levels, to appear insignificant due to under-representation, despite their expected effect on C_dyn_. Further study is required to determine their possible effects on models used to calculate C_dyn_.

Fifthly, the absence of preoperative imaging studies to assess for underlying respiratory diseases may limit the interpretation of the results, as undetected respiratory conditions could influence C_dyn_ measurements.

Finally, the diagnostic tests performed on our multiple linear regression model yielded mixed results. The assumption of linearity was confirmed, indicating that the relationship between the predictor variables and the response variable was appropriately modeled as linear. Additionally, the independence of residuals was verified, suggesting that there are no significant autocorrelation issues. Importantly, multicollinearity was not a concern, ensuring that the predictor variables are not highly correlated, and the regression coefficients remain stable. However, the tests indicated violations in both homoscedasticity and the normality of residuals. These findings suggest that the variance of the residuals is not constant, and that the residuals do not follow a normal distribution, respectively. These violations may affect the efficiency and validity of our model's estimates and hypothesis tests. To address potential bias in the linear regression coefficient estimates, bootstrapping was applied, and confidence intervals were calculated. This approach is commonly used to provide more robust estimates and mitigate the impact of violations in residual normality. Furthermore, due to the presence of heteroscedasticity, robust standard errors were calculated, and *p*-values accordingly adjusted. While caution is warranted when extrapolating the presented results, the application of these calculations has yielded more reliable estimates for further inference.

## 5 Conclusion

Many veterinary anesthetists employ P-V loops and specifically monitor respiratory system compliance (1). This study revealed that C_dyn_ should be evaluated relative to body mass. While a definitive reference interval was not established and may lack clinical utility, the study demonstrated that C_dyn_ was significantly influenced by several factors: the internal diameter of orotracheal tubes, BCS, inspiratory time settings, the specific sensor used for C_dyn_ determination, and exposure to FIO_2_ levels exceeding 80% for at least 10 minutes before assessment. Minimal inspiratory setting characteristics (such as inspiratory time, or inspiratory-to-expiratory ratio and set respiratory rate) should be considered when clinically comparing C_dyn_ over time and systematically reported in research.

## Data Availability

The datasets presented in this study can be found in online repositories. The names of the repository/repositories and accession number(s) can be found at: The datasets analyzed for this study can be found in the BORIS (Bern Online Repository and Information System (https://boris-portal.unibe.ch/handle/20.500.12422/33611).
