# Supplementary material for: Characterization of dynamic compliance of the respiratory system in healthy anesthetized dogs
Source: Front Vet Sci. 2024 Nov 28;11:1490494. doi: 10.3389/fvets.2024.1490494 (PMC11634835; doi:10.3389/fvets.2024.1490494)
Supplement: Supplementary file 1 [file Data_Sheet_1.pdf]

Supplementary Material

Characterization of dynamic compliance of the respiratory system in anaesthetized dogs

Mathieu Raillard*, Martina Mosing, Anthea Raisis, Adam Auckburally, Georgina Beaumont, Frances Downing, Charlotte Heselton, Paul MacFarlane, Karine Portier, Josephine Robertson, Joao Henrique Neves Soares, Barbara Steblaj, Elliot Wringe, Olivier L. Levionnois

*** Correspondence:**
Olivier L. Levionnois
olivier.levionnois@unibe.ch

1 Characterization of dynamic compliance (C_{dyn})

- Shapiro-Wilk normality test, W = 0.918, *p*-value <0.001 (rejects normal distribution)
- Anderson-Darling normality test, A = 11.99, p-value <0.001 (rejects normal distribution)

Supplementary Figure 1. Frequency histogram of C_{dyn} including the median (dashed line) and 75% interquartiles (dotted lines) in a cohort of 515 dogs anaesthetized in 11 centres across six countries. Anaesthetic management was at the discretion of the local anaesthesia team, and the dogs were undergoing clinical procedures based on their individual conditions.

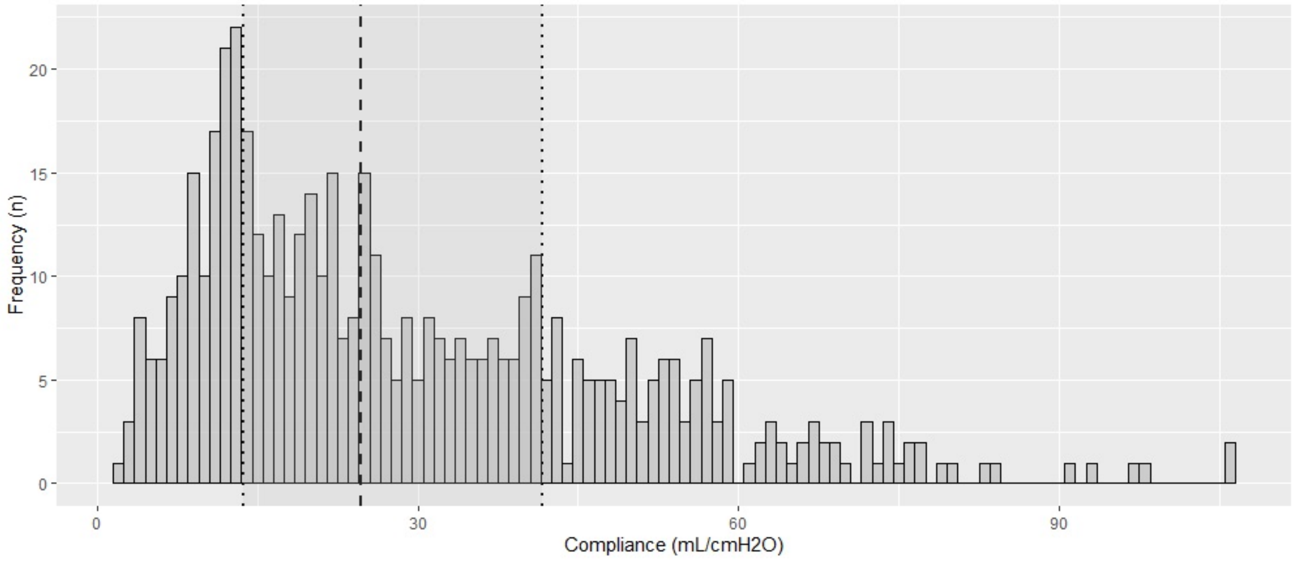

2 Linear models of dynamic compliance (C_{dyn}) against body mass (BM)

- n = 503 dogs, BM = **20.4** ± 12.6 kg, Quartiles [1.5-10.8-**18.2**-28.1-86]

Linear model crossing origin:

- Comparison to Asorey et al. (2020): C_{RS} = 1.3 ± 0.3 mL/cmH_2O, BM [10-35] kg *(C_{RS}: compliance of the respiratory system, abbreviation used in the original research)*
- lm(formula = Compliance ~ 0 + Mass, data = data)

Supplementary Table 1A. Linear regression of C_{dyn} against body mass with free intercept in a cohort of 515 dogs anaesthetized in 11 centres across six countries. Anaesthetic management was at the discretion of the local anaesthesia team, and the dogs were undergoing clinical procedures based on their individual conditions.

	**Estimate**	**Std. Error**	***p***
Mass	1.411	0.0215	<0.001
**Adjusted R^2:** 0.893			

Linear model with intercept:

- Comparison to Bradbrook et al. (2013): Crs = 0.895 × BM + 8.845 *(C_{RS}: compliance of the respiratory system, abbreviation used in the original research)*
- lm(formula = Compliance ~ Mass, data = data)

Supplementary Table 1B. Linear regression of C_{dyn} against body mass with intercept in a cohort of 515 dogs anaesthetized in 11 centres across six countries. Anaesthetic management was at the discretion of the local anaesthesia team, and the dogs were undergoing clinical procedures based on their individual conditions.

	**Estimate**	**Std. Error**	***p***
Intercept	3.672	0.987	<0.001
Mass	1.284	0.041	<0.001
**Adjusted R^2:** 0.656			

Comparison of the linear models with and without intercept:

Supplementary Table 1C. Comparison of the best fitting linear regressions of C_{dyn} against body mass with and without intercept in a cohort of 515 dogs anaesthetized in 11 centres across six countries. Anaesthetic management was at the discretion of the local anaesthesia team, and the dogs were undergoing clinical procedures based on their individual conditions.

**ANOVA**, $p < 0.001$	AIC	BIC
Model without intercept	3909.721	3918.162
Model with intercept	3898.019	3910.681

3 Relationship between body mass (BM) and internal diameter of the orotracheal tubes (ETT_ID)

- Relationship between ETT_ID and BM:
  - Literature: (Shin et al. 2018; Tong & Pang 2019 ; Haider et al. 2020)
    - Shin et al. (2018): ETT_ID = 70% of trachea diameter
    - Tong at al. (2019): ETT_ID = Cube root of body mass
    - Haider et al. (2020): ETT_ID = 2.4*ln(Body mass)+1.8
  - Models fitted following a logarithmic increase followed by a plateau:
    - **<u>Root</u>** $a \times \left(b - \sqrt{x + c}\right) + d$
      - [**NEG(c)**=BM_Min (0-5), d=ETT_ID_Min (2-3.5)]
      - b [1-4]; 1 = linear
    - **<u>Ln</u>** $(a \ln x)^b + d$
      - [**NEG(c)**=BM_Min (0-5), d=ETT_ID_Min (2-3.5)]
      - b [0.1-1.9]; >1.9 = sigmoid
    - **<u>Emax model</u>** $a \times \frac{x^b}{c^b + x^b} + d$
      - [d=ETT_ID_Min (2-3.5), d+a=ETT_ID_Max]
      - c=EM_50% of ETT_ID

Supplementary Table 2. Root, Ln and Emax models and their comparison to evaluate the relationship between internal diameter of the orotracheal tubes and body mass in 494 dogs anaesthetized in 11 centres across six countries (nine cases were removed from the analysis, two for missing values and seven for suspicion of incorrect values). Anaesthetic management was at the discretion of the local anaesthesia team, and the dogs were undergoing clinical procedures based on their individual conditions.

Model	a	b	c	d	AIC	BIC
**Root**	1.253	1.853	-2.55	3.5	1767.816	1788.829
**Ln**	0.74	1.9		3.5	1748.754	1765.564
**Emax**	13.459	1.244	23.612	3.5	1735.364	1756.377

Supplementary Figure 2. Internal diameters of orotracheal tubes (in mm) against body mass of 494 dogs anaesthetized in 11 centres across six countries (nine cases were removed from the analysis, two for missing values and seven for suspicion of incorrect values). Anaesthetic management was at the discretion of the local anaesthesia team, and the dogs were undergoing clinical procedures based on their individual conditions. The three best-fitting models are represented: a root function (model 1, continuous line), a logarithmic function (model 2, dashed line), and a sigmoid Emax model (model 3, dotted line).

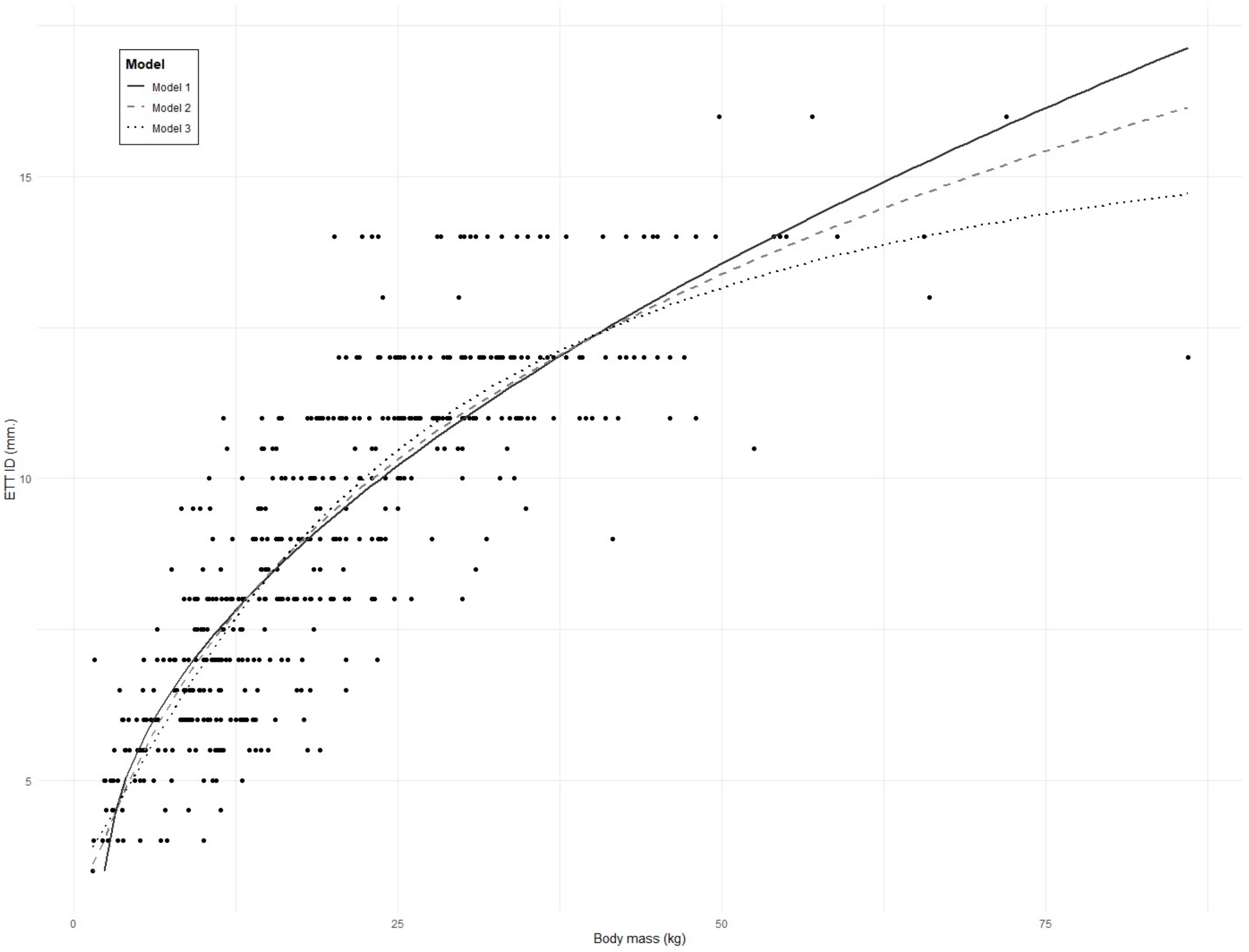

References

Haider G, Lorinson K, Lorinson D, Auer U. Development of a clinical tool to aid endotracheal tube size selection in dogs. *Vet Rec*. (2020) 186:157. doi: 10.1136/vr.105065

Shin CW, Son WG, Jang M et al. Changes in endotracheal tube intracuff pressure and air leak pressure over time in anesthetized Beagle dogs. *Vet Anaesth Analg.* (2018) 45**,** 737-744. Doi: 10.1016/j.vaa.2018.06.005

Tong J & Pang DSJ. Investigating novel anatomical predictors for endotracheal tube selection in dogs. *Can Vet J*. (2019) 60**,** 848-854.

4 Regression model for dynamic compliance (C_{dyn}) against body mass including orotracheal tubes internal diameter (ETT_ID) categories

- ETT_ID categories were created by evaluating the ratio of the difference between actual and predicted ETT_ID values to the predicted values:
  - ([ETT_ID_Actual] – [ETT_ID_Predicted]) / [ETT_ID_Predicted]
  - Best cut-off: two categories:
    - "Small": < -7% of predicted ETT_ID
    - "Medium/large": > -7% of predicted ETT_ID

Supplementary Figure 3. Internal diameters of orotracheal tubes (ETT_ID, in mm) against body mass of 494 dogs anaesthetized in 11 centres across six countries, including the prediction curve from the best fitting sigmoid Emax model (continuous line), and the groups "Small" (gray filling, ETT_ID below 7% smaller than the predicted value) and "Medium/large" (white filling, ETT_ID above 7% smaller than the predicted value).

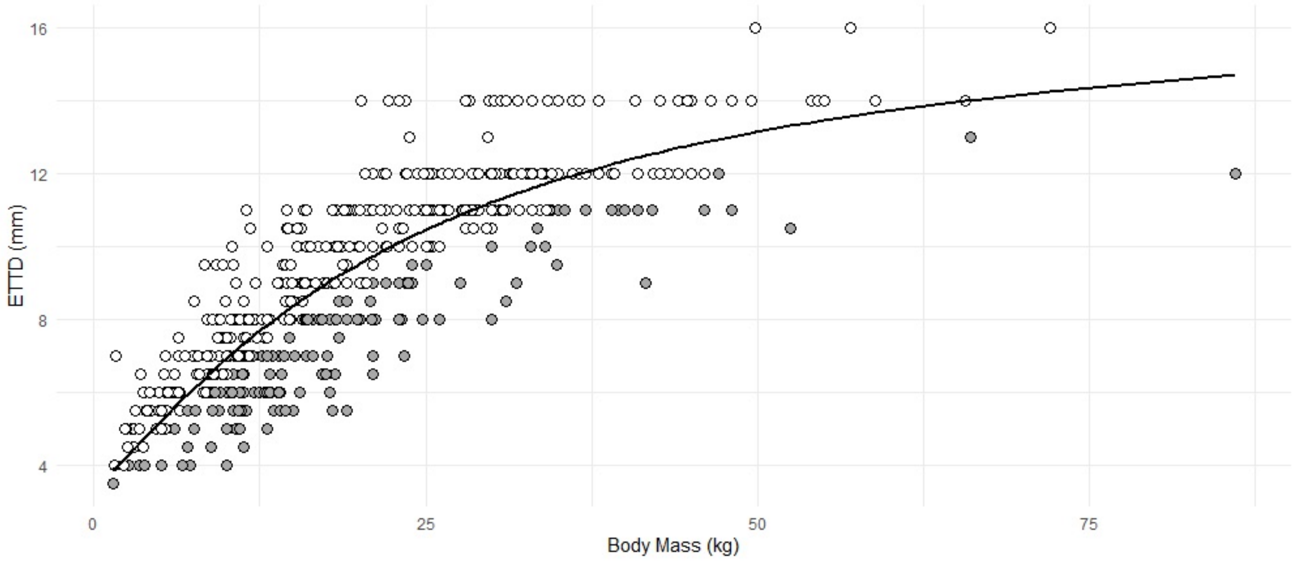

5 Characterization and effects of age

- n = 487
- No linear relationship between C_{dyn} and Age (p = 0.556, adjusted R^2 = -0.0013)
- No difference of Age inclusion in the C_{dyn}/Body mass model (p =0.347)
  - Adjusted R^2 unchanged (0.654 -> 0.654)
  - AIC increased (3776.622 -> 3777.732)
  - BIC increased (3789.186 -> 3794.485)

Supplementary Figure 4A. Frequency histogram of age (in months) of 487 dogs anaesthetized in 11 centres across six countries. Anaesthetic management was at the discretion of the local anaesthesia team, and the dogs were undergoing clinical procedures based on their individual conditions.

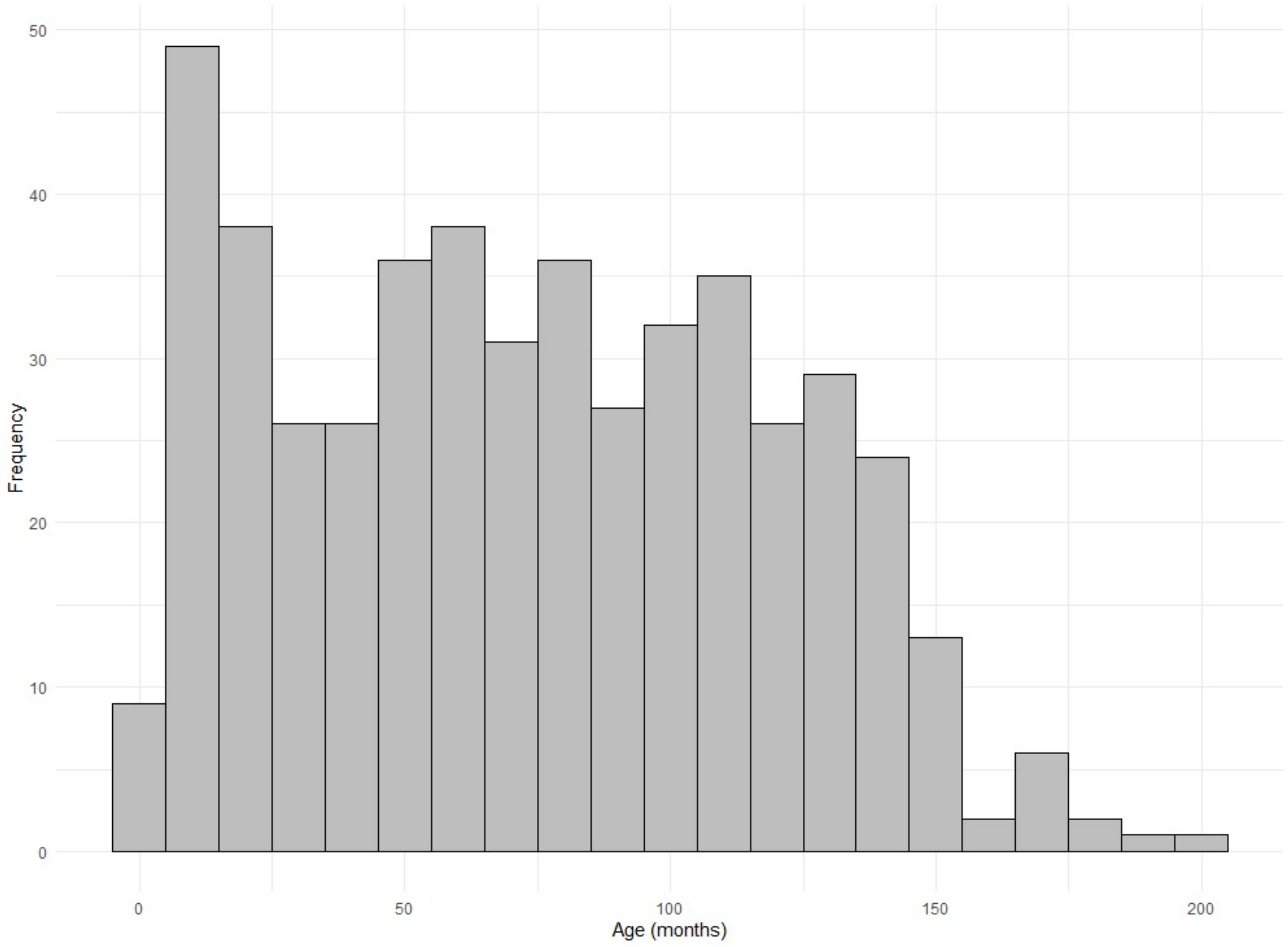

Supplementary Figure 4B. Best-fitting linear regression of C_{dyn} against age in a cohort of 487 dogs anaesthetized in 11 centres across six countries. Anaesthetic management was at the discretion of the local anaesthesia team, and the dogs were undergoing clinical procedures based on their individual conditions.

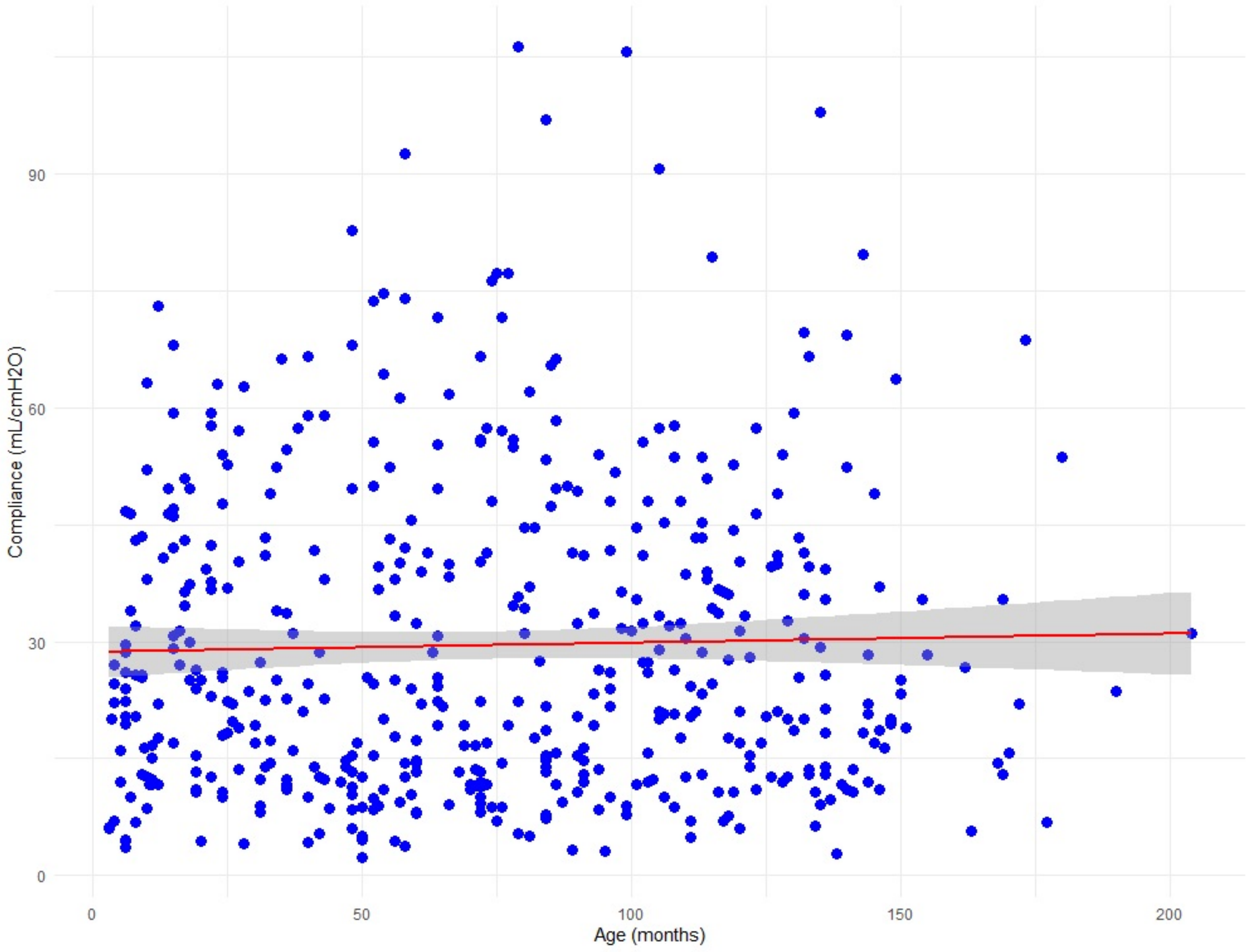

6 Characterization and effects of Body Condition Score (BCS)

- n = 491

Supplementary Figure 5A. Frequency histogram of Body Condition Score (BCS, from 1: very thin, to 9: obesity) of 491 dogs anaesthetized in 11 centres across six countries. Anaesthetic management was at the discretion of the local anaesthesia team, and the dogs were undergoing clinical procedures based on their individual conditions.

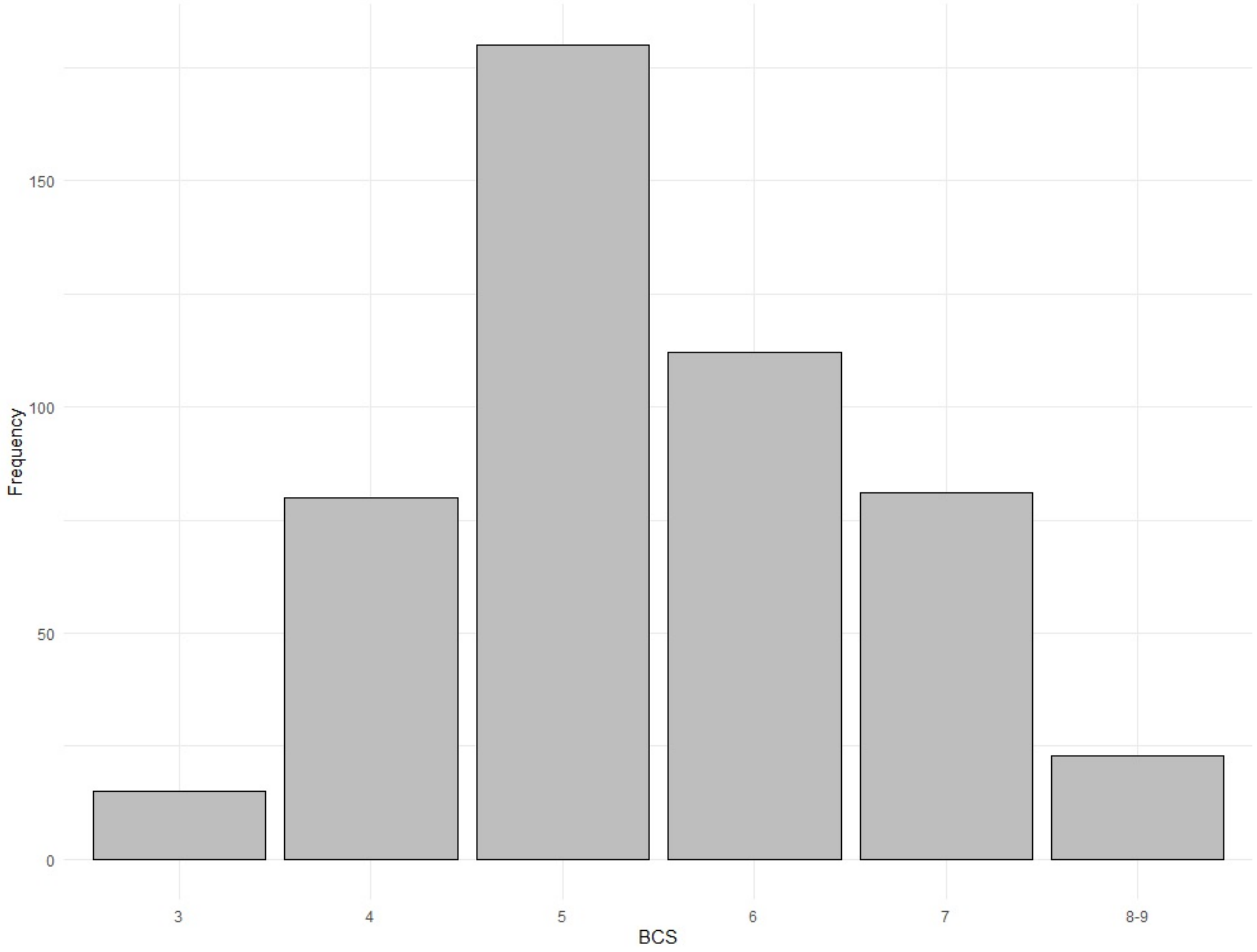

Supplementary Figure 5B. Box plots (median, interquartiles, range, outsiders) of dynamic compliance (C_{dyn}) for each category of BCS score (from 1: very thin, to 9: obesity) of 491 dogs anaesthetized in 11 centres across six countries. Anaesthetic management was at the discretion of the local anaesthesia team, and the dogs were undergoing clinical procedures based on their individual conditions.

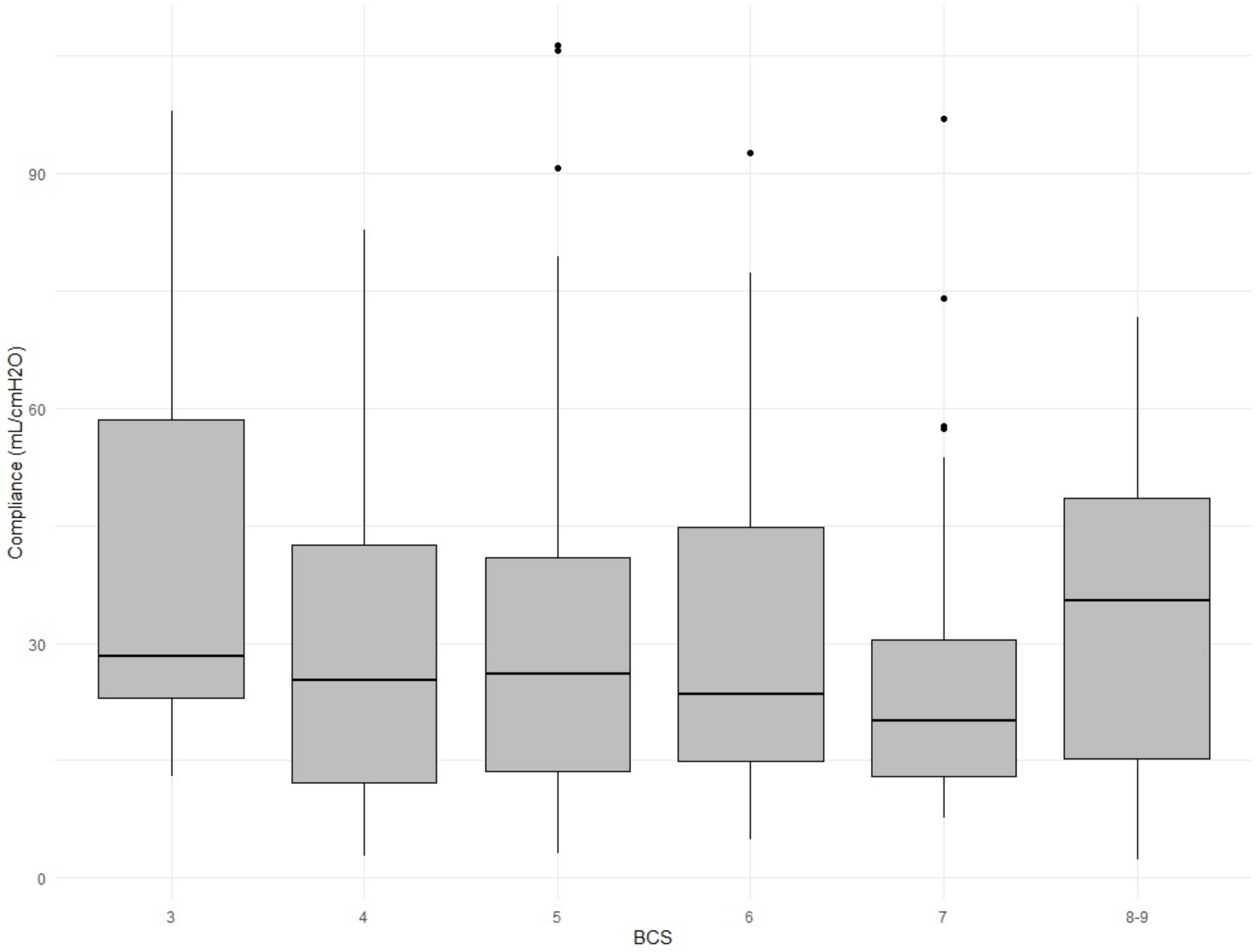

Supplementary Figure 5C. Box plots (median, interquartiles, range, outsiders) of dynamic compliance (C_{dyn}) for the categories of low BCS (score 1-3), medium BCS (score 4-5), and high (score 6-9) of 491 dogs anaesthetized in 11 centres across six countries. Anaesthetic management was at the discretion of the local anaesthesia team, and the dogs were undergoing clinical procedures based on their individual conditions.

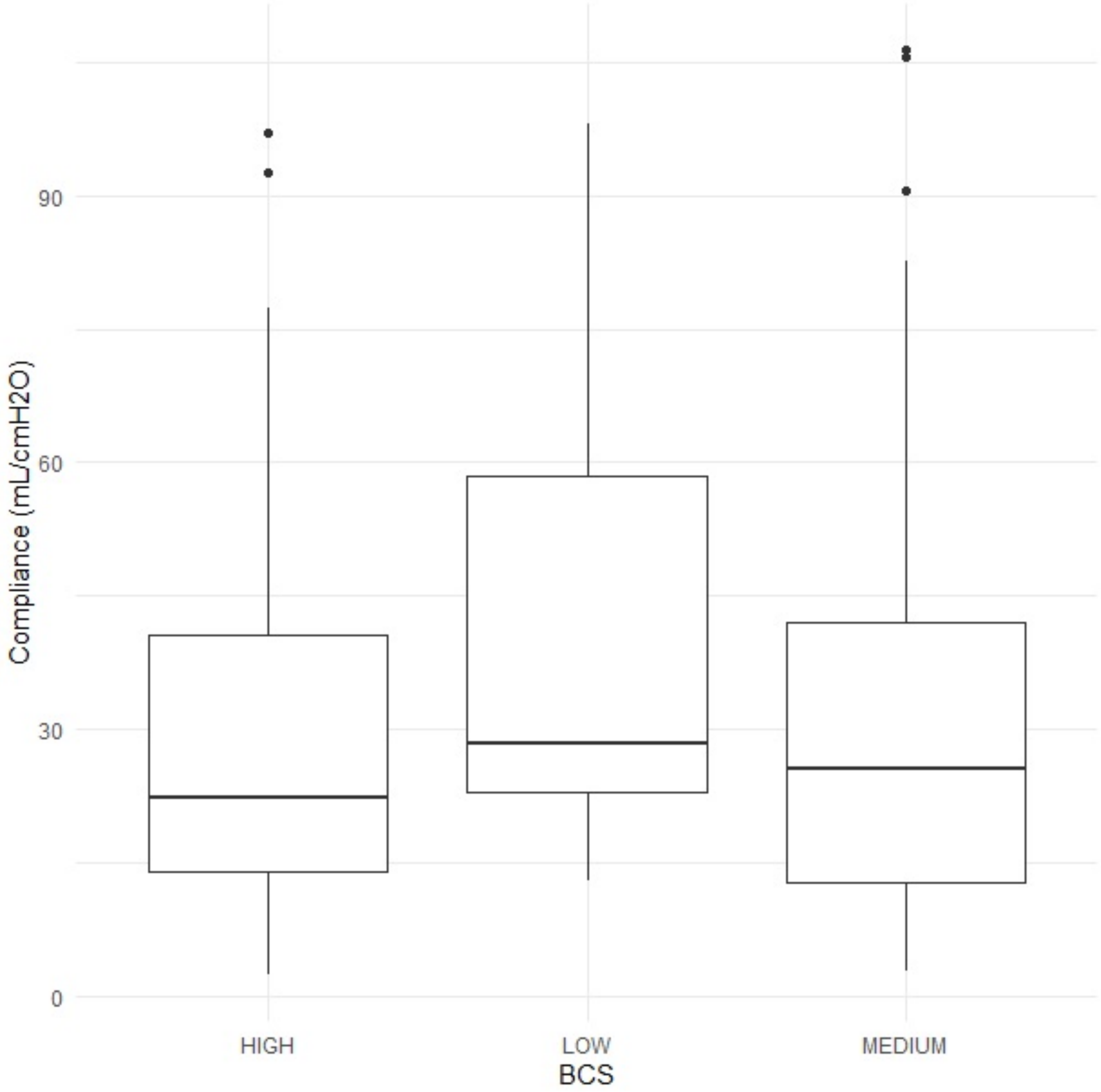

7 Characterization and effects of Morphology (Dolichomorphic, Brachymorphic, Mesomorphic)

- n = 494
- Kruskal-Wallis rank sum test for C_{dyn} among Morphology:
    - $Chi^2 = 35.272$, degree of freedom = 2, *p*-value <0.001

Supplementary Figure 6A. Frequency histogram of morphology of 494 dogs anaesthetized in 11 centres across six countries. Anaesthetic management was at the discretion of the local anaesthesia team, and the dogs were undergoing clinical procedures based on their individual conditions.

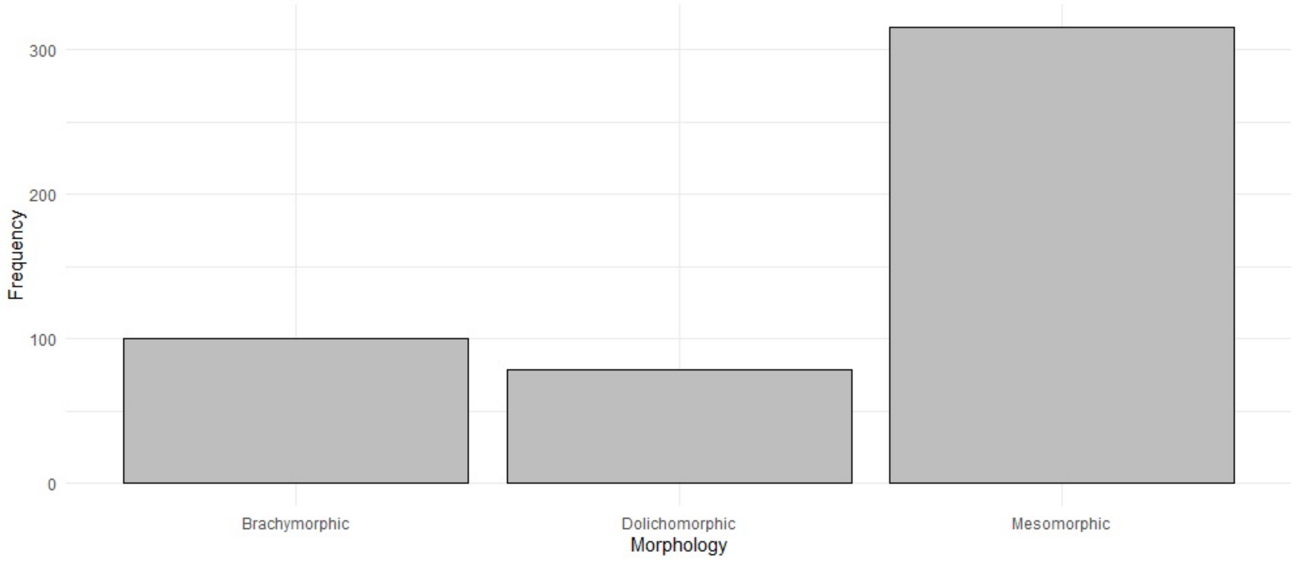

Supplementary Figure 6B. Box plots (median, interquartiles, range, outsiders) of dynamic compliance (C_{dyn}) for each category of morphology (brachymorphic, dolichomorphic, mesomorphic) of 491 dogs anaesthetized in 11 centres across six countries. Anaesthetic management was at the discretion of the local anaesthesia team, and the dogs were undergoing clinical procedures based on their individual conditions.

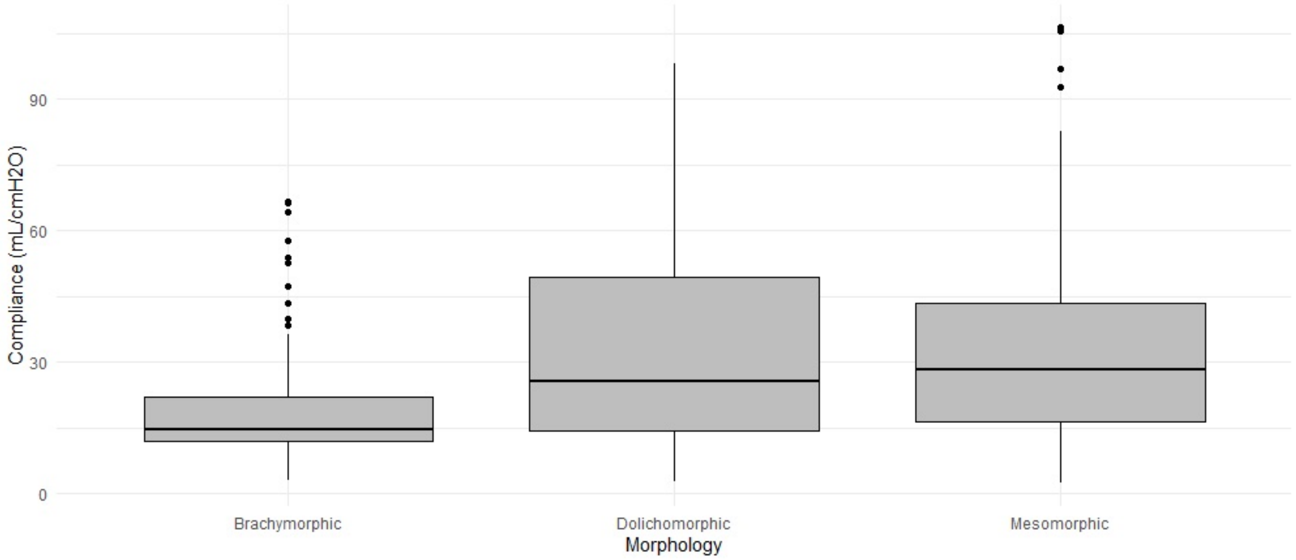

8 Characterization and effects of Position (Sternal vs Dorsal vs Lateral)

- n = 492
- Kruskal-Wallis rank sum test for C_{dyn} among Position:
  - Chi^2 = 10.101, degree of freedom = 2, *p*-value = 0.006
  - The position influences C_{dyn}
- No Effect of Position inclusion in the C_{dyn}/Body mass model (p = 0.096)
  - Adjusted R^2 slightly increased (0.655 -> 0.657)
  - AIC unchanged (3815.75 -> 3815.03)
  - BIC increased (3828.35 -> 3836.02)
- Wilcoxon rank-sum test for C_{dyn} among Lateral position vs other position:
  - *p*-value = 0.003

Supplementary Figure 7A. Frequency histogram of position of 492 dogs anaesthetized in 11 centres across six countries. Anaesthetic management was at the discretion of the local anaesthesia team, and the dogs were undergoing clinical procedures based on their individual conditions.

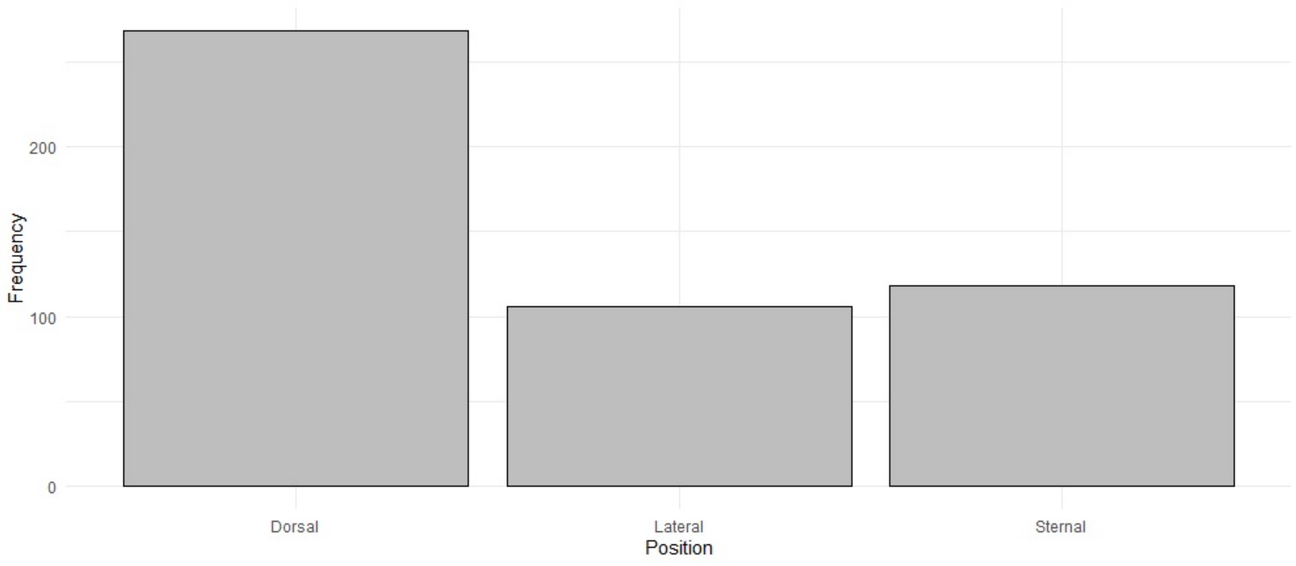

Supplementary Figure 7B. Box plots (median, interquartiles, range, outsiders) of dynamic compliance (C_{dyn}) according to lateral recumbency vs non (i.e., sternal or dorsal recumbency) of 492 dogs anaesthetized in 11 centres across six countries. Anaesthetic management was at the discretion of the local anaesthesia team, and the dogs were undergoing clinical procedures based on their individual conditions.

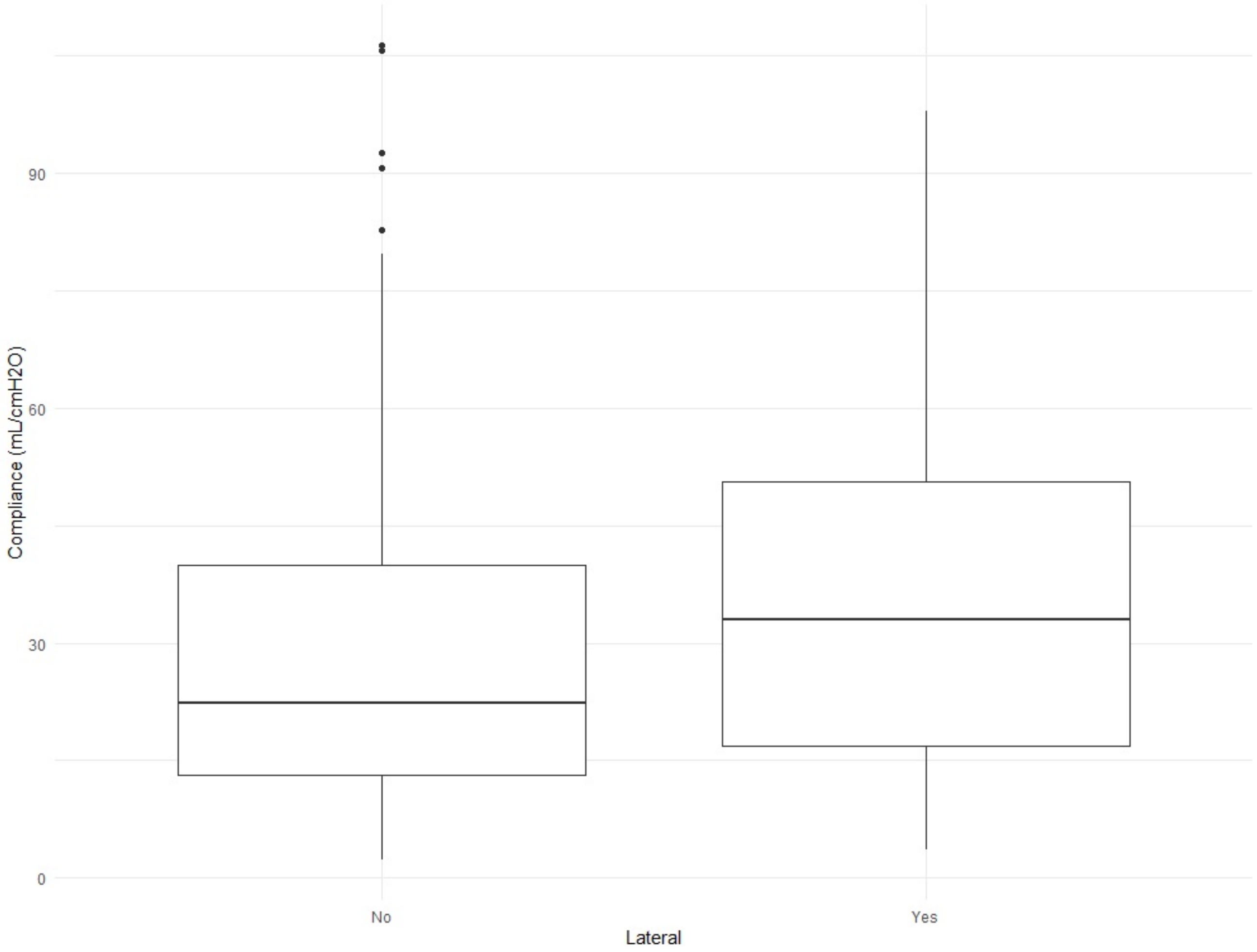

9 Characterization and effects of the sensor selection (Pedi-lite vs D-lite vs Other, ventilator's in-built spirometry sensor)

- $n = 491$
- Kruskal-Wallis rank sum test for C_{dyn} among Sensors:
  - $Chi^2 = 181.54$, degree of freedom = 2, p-value <0.001

Supplementary Figure 8A. Frequency histogram of the sensors (Pedi-lite, D-lite, other ventilator's in-built spirometry sensor) used to establish dynamic compliance (C_{dyn}) of 491 dogs anaesthetized in 11 centres across six countries. Anaesthetic management was at the discretion of the local anaesthesia team, and the dogs were undergoing clinical procedures based on their individual conditions.

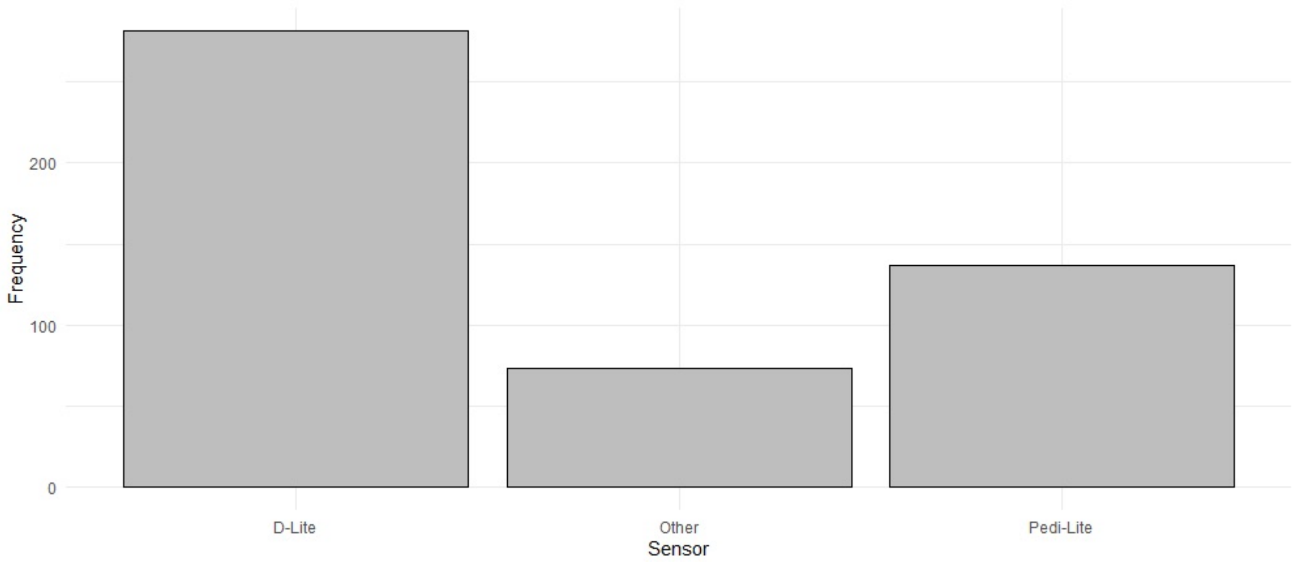

Supplementary Figure 8B. Box plots (median, interquartiles, range, outsiders) of dynamic compliance (C_{dyn}) according to the sensors used to establish C_{dyn} (Pedi-lite, D-lite, any other ventilator's in-built spirometry sensor) in 491 dogs anaesthetized in 11 centres across six countries. Anaesthetic management was at the discretion of the local anaesthesia team, and the dogs were undergoing clinical procedures based on their individual conditions.

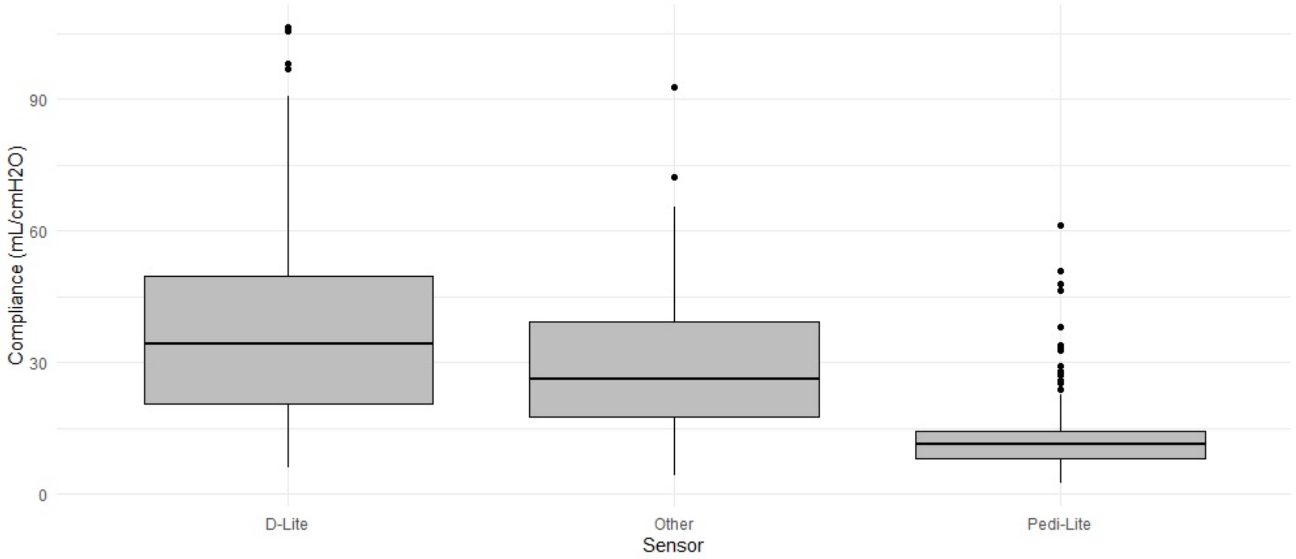

10 Characterization and effects of the presence or absence of Heat and Moisture Exchangers (HMEs)

- n = 490
- Although some HMEs were placed at the junction between expiratory limb of the breathing system and expiratory valve, they were treated as HME not present from a C_{dyn} point of view; Wilcoxon rank-sum test for C_{dyn} the presence or absence of HMEs:
    - *p*-value = 0.487
- No difference of HME inclusion in the C_{dyn} /Body mass model ($p = 0.103$)
    - Adjusted R^2 increased (0.655 -> 0.654)
    - AIC slightly decreased (3794.26 -> 3793.58)
    - BIC slightly decreased (3806.83 -> 3810.35)

Supplementary Figure 9A. Frequency histogram of Heat and Moisture Exchangers (HMEs; none or present at the dog's end) in 490 dogs anaesthetized in 11 centres across six countries. Anaesthetic management was at the discretion of the local anaesthesia team, and the dogs were undergoing clinical procedures based on their individual conditions.

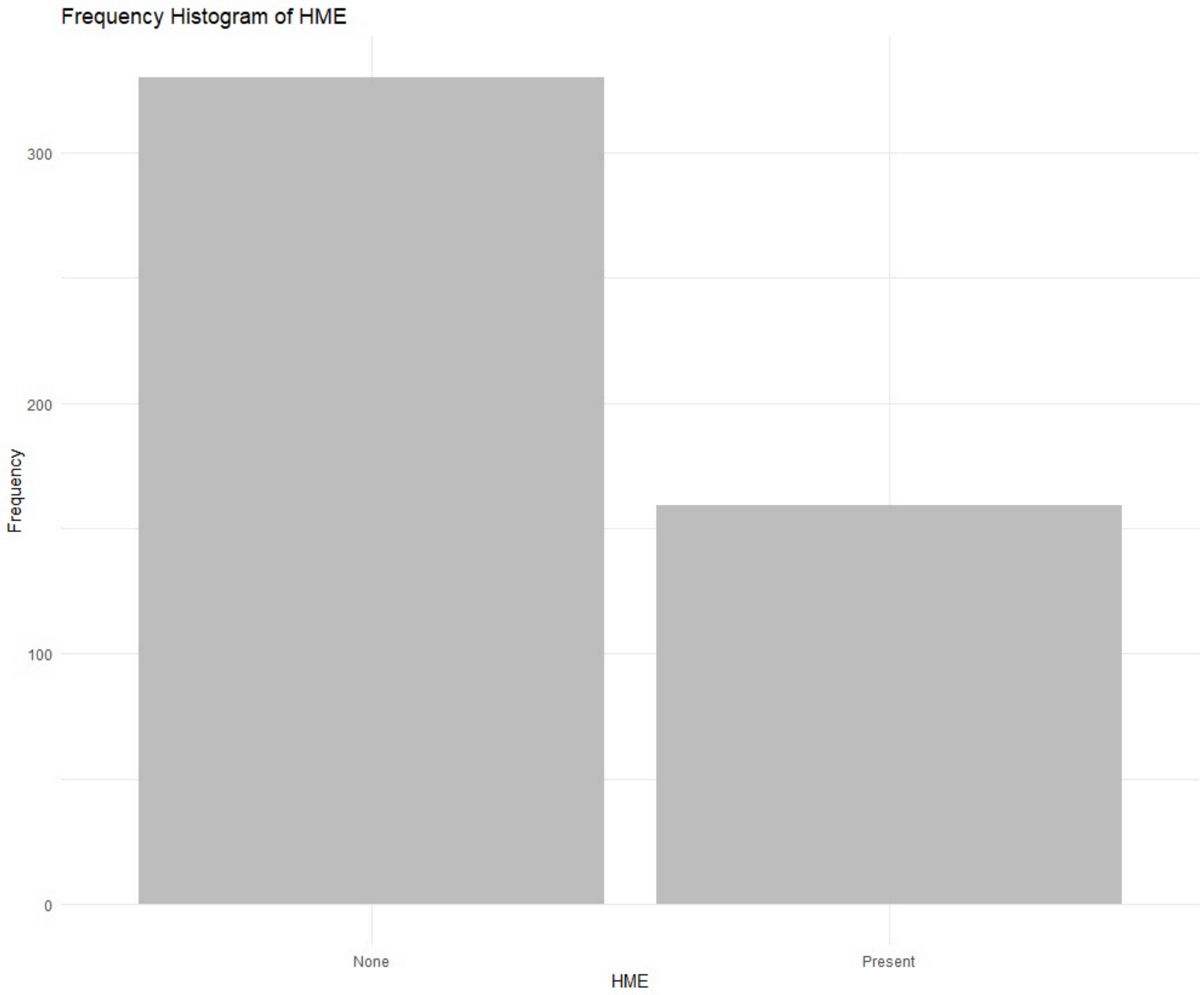

Supplementary Figure 9B. Box plots (median, interquartiles, range, outsiders) of dynamic compliance (C_{dyn}) according to the use of Heat and Moisture Exchangers (HMEs; none or present at the dog's end) in 491 dogs anaesthetized in 11 centres across six countries. Anaesthetic management was at the discretion of the local anaesthesia team, and the dogs were undergoing clinical procedures based on their individual conditions.

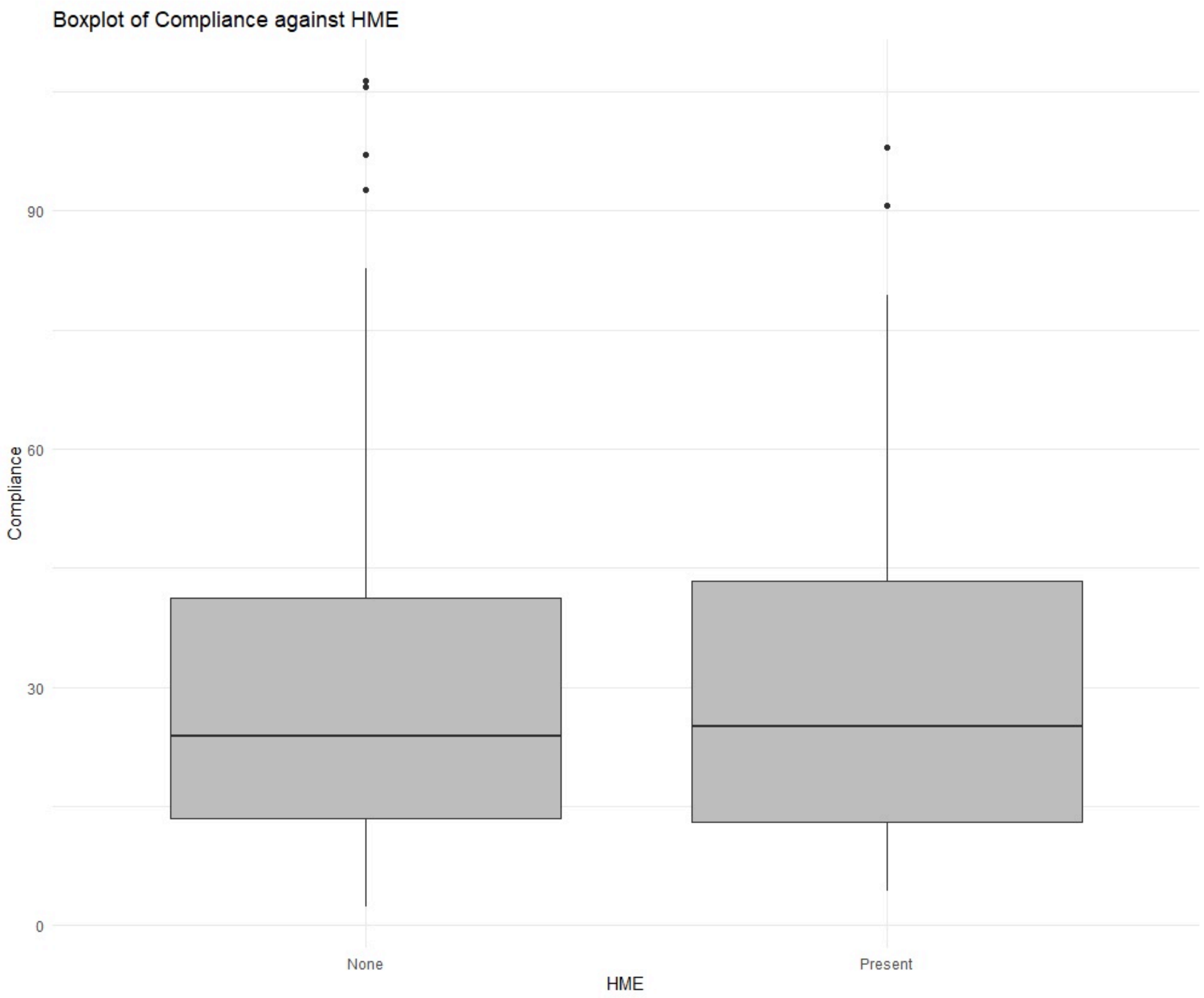

Supplementary Figure 9C. Best-fitting linear regression of C_{dyn} against body mass according to the use of Heat and Moisture Exchangers: none (black triangles, continuous line), or present (black circles, dashed line) in a cohort of 491 dogs anaesthetized in 11 centres across six countries. Anaesthetic management was at the discretion of the local anaesthesia team, and the dogs were undergoing clinical procedures based on their individual conditions.

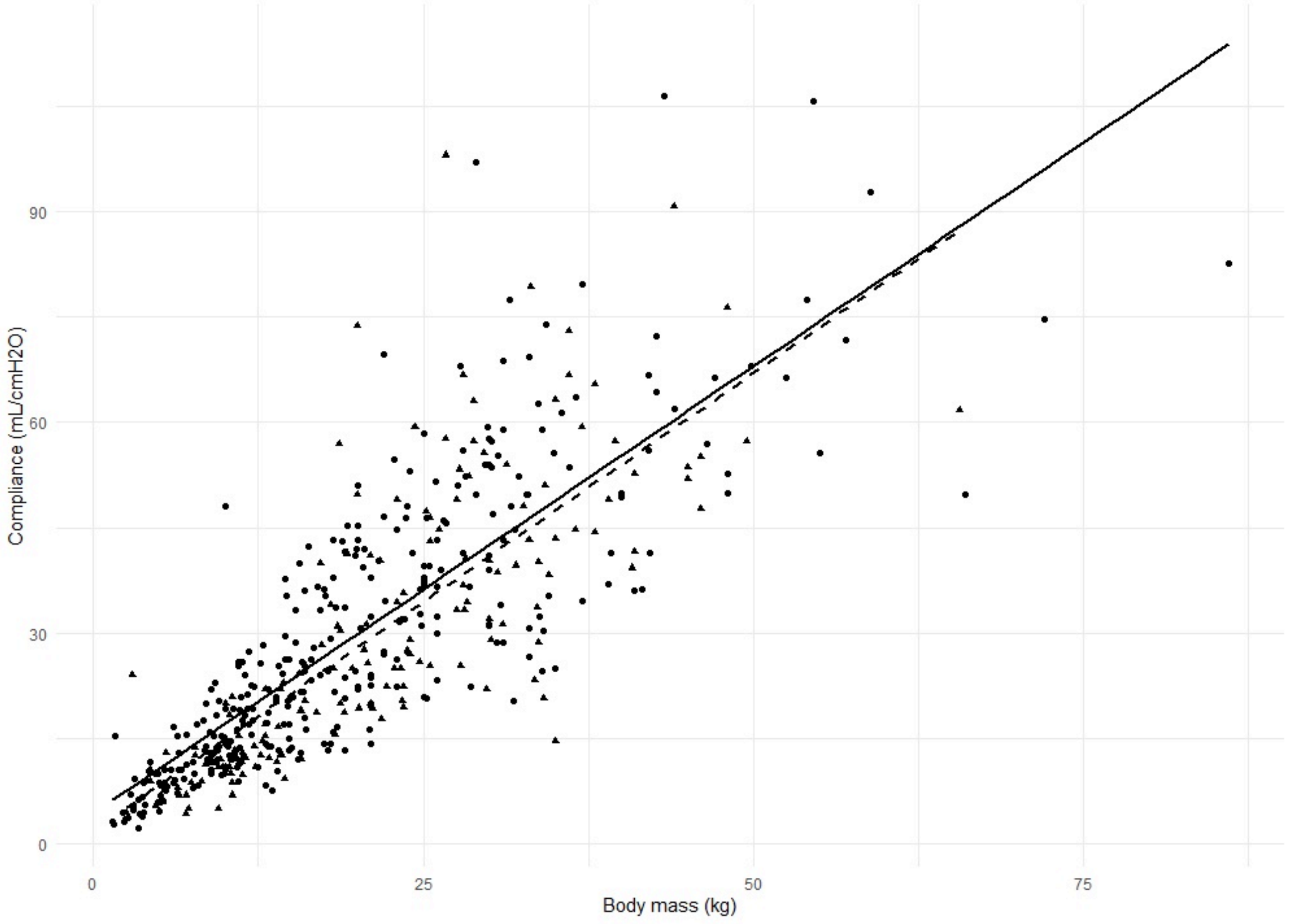

11 Characterization and effects of the inspired fraction of oxygen (FIO_2)

- n = 487
- No linear relationship between C_{dyn} and FIO_2 ($p = 0.149$, adjusted $R^2 = 0.002$)
- No difference of FIO_2 inclusion in the C_{dyn}/Body mass model ($p = 0.151$):
    - Adjusted R^2 unchanged (0.654 -> 0.655)
    - AIC unchanged (3779.435 -> 3779.363)
    - BIC slightly increased (3792 -> 3796.116)

Supplementary Figure 10A. Frequency histogram of the inspired fraction of oxygen (FIO_2, in %) in 487 dogs anaesthetized in 11 centres across six countries. Anaesthetic management was at the discretion of the local anaesthesia team, and the dogs were undergoing clinical procedures based on their individual conditions.

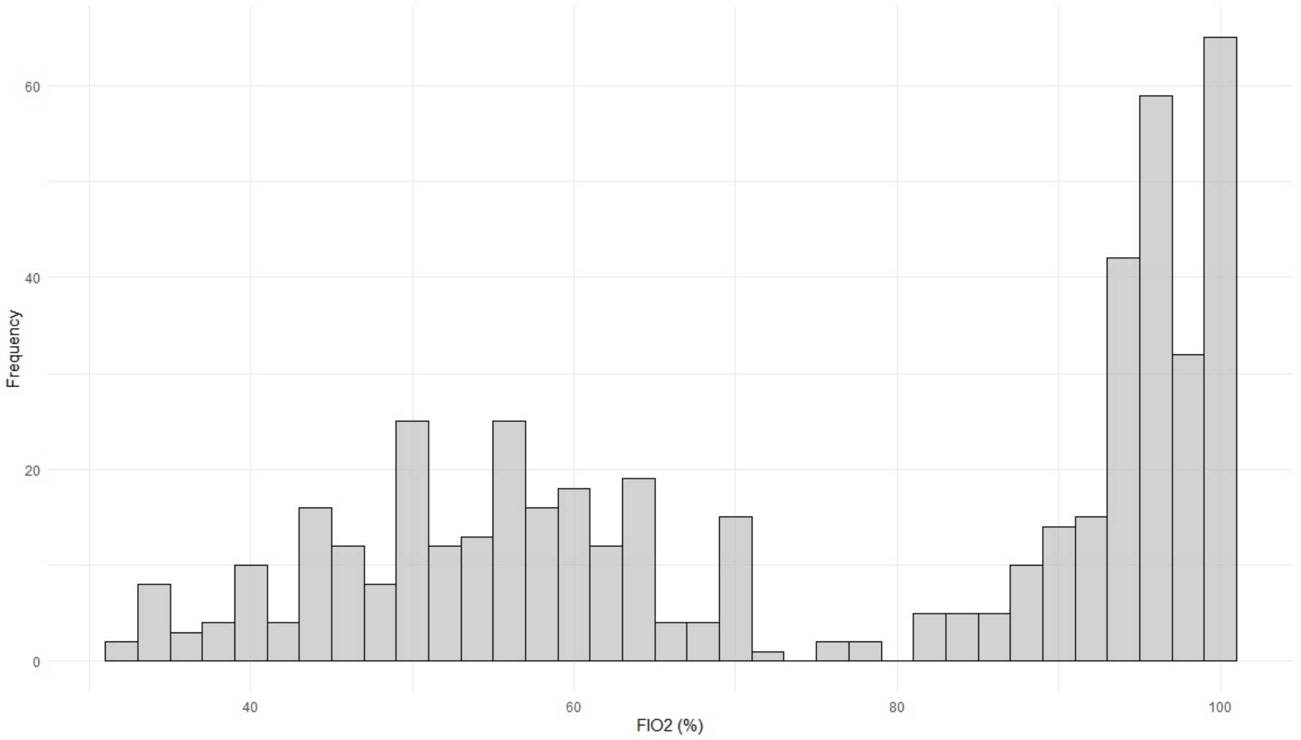

Supplementary Figure 10B. Best-fitting linear regression of C_{dyn} against the inspired fraction of oxygen (FIO_2, in %) in a cohort of 487 dogs anaesthetized in 11 centres across six countries. Anaesthetic management was at the discretion of the local anaesthesia team, and the dogs were undergoing clinical procedures based on their individual conditions.

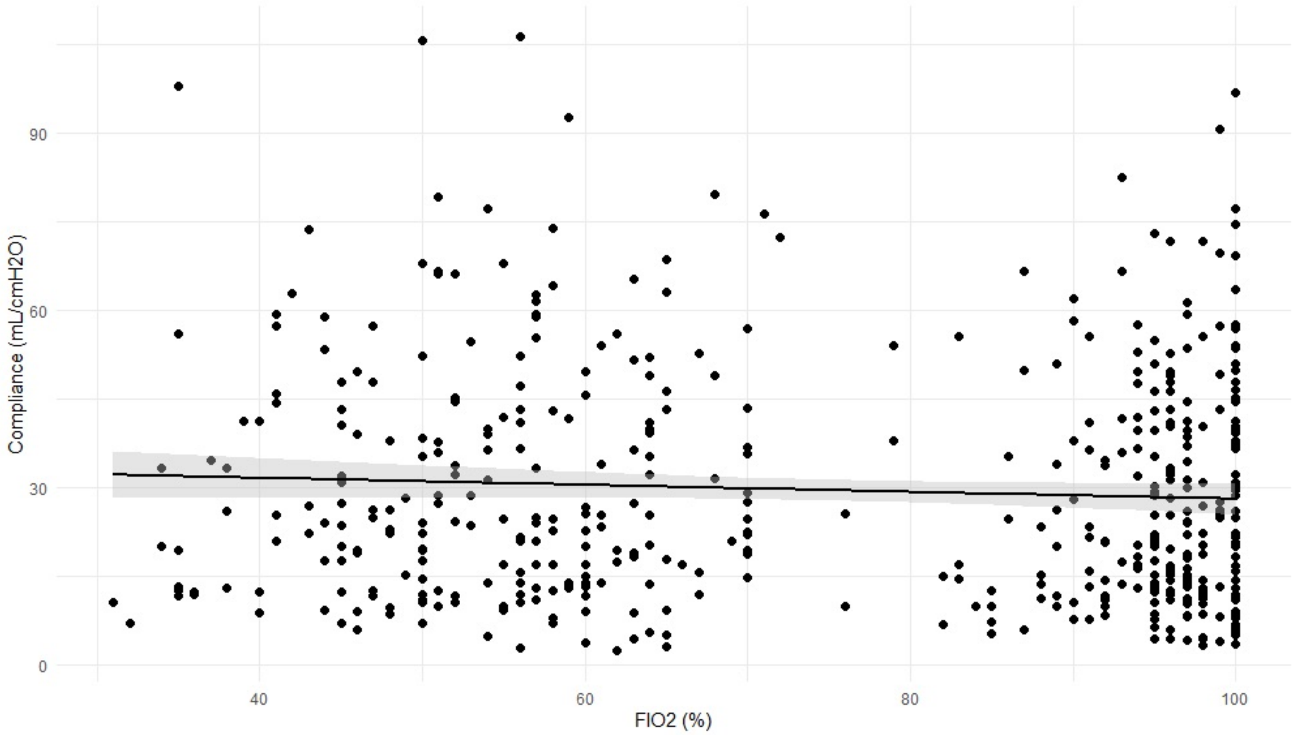

12 Characterization and effects of an inspired fraction of oxygen (FIO_2) above 80% for at least 10 minutes before the measurements

- n = 492
- Wilcoxon rank-sum test for C_{dyn} in cases having been administered a FIO_2>80% for at least 10 minutes before the measurement vs not:
  - *p*-value = 0.002

Supplementary Figure 11A. Frequency histogram (Yes) or not (No) of dogs being administered a FIO_2>80% for at least 10 minutes before determining the dynamic compliance (C_{dyn}). The population enrolled consisted in 492 dogs anaesthetized in 11 centres across six countries. Anaesthetic management was at the discretion of the local anaesthesia team, and the dogs were undergoing clinical procedures based on their individual conditions.

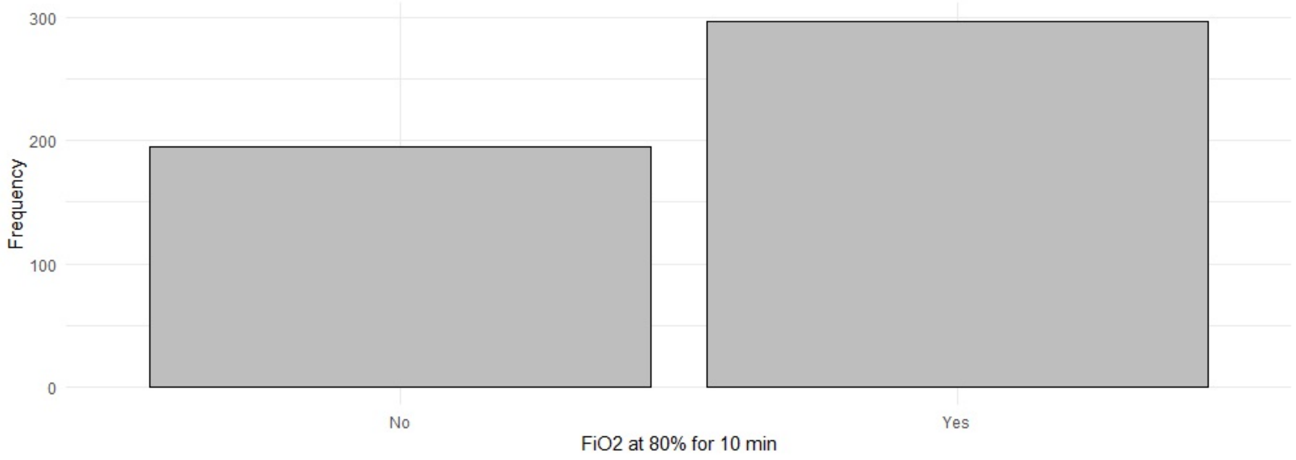

Supplementary Figure 11B. Box plots (median, interquartiles, range, outsiders) of dynamic compliance (C_{dyn}) according to the administration or not of a FIO_2>80% for at least 10 minutes before the clinical measurement in 492 dogs anaesthetized in 11 centres across six countries. Anaesthetic management was at the discretion of the local anaesthesia team, and the dogs were undergoing clinical procedures based on their individual conditions.

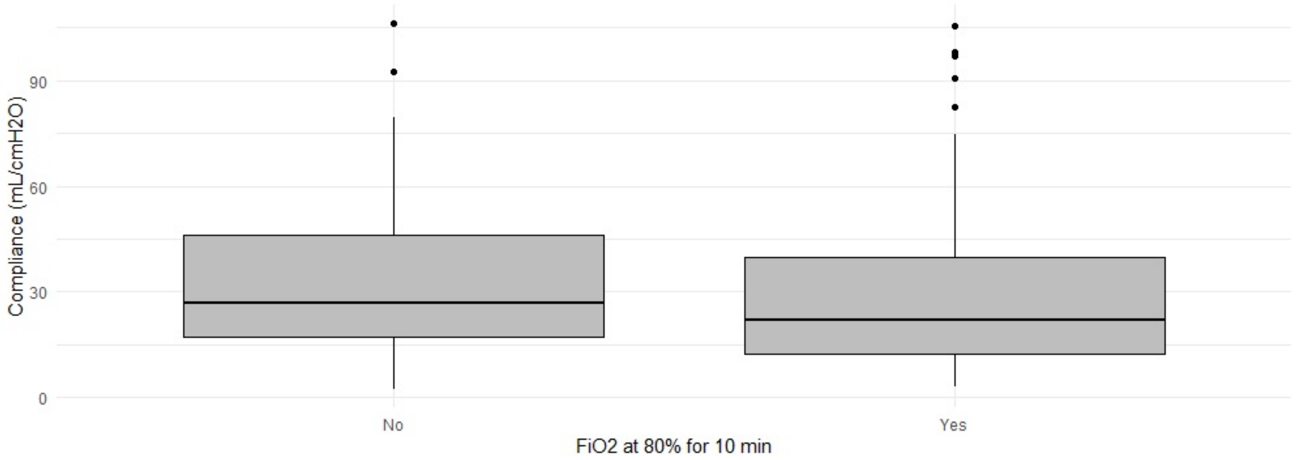

13 Characterization and effects of the time between induction of anaesthesia and the measurements

- n = 484
- Weak linear relationship between C_{dyn} and time post induction
  - $p = 0.05$, Adjusted $R^2 = 0.006$

Supplementary Figure 12. Frequency histogram of the time (in minutes) between induction of general anaesthesia and the determination of the dynamic compliance (C_{dyn}) in 484 dogs anaesthetized in 11 centres across six countries. Anaesthetic management was at the discretion of the local anaesthesia team, and the dogs were undergoing clinical procedures based on their individual conditions.

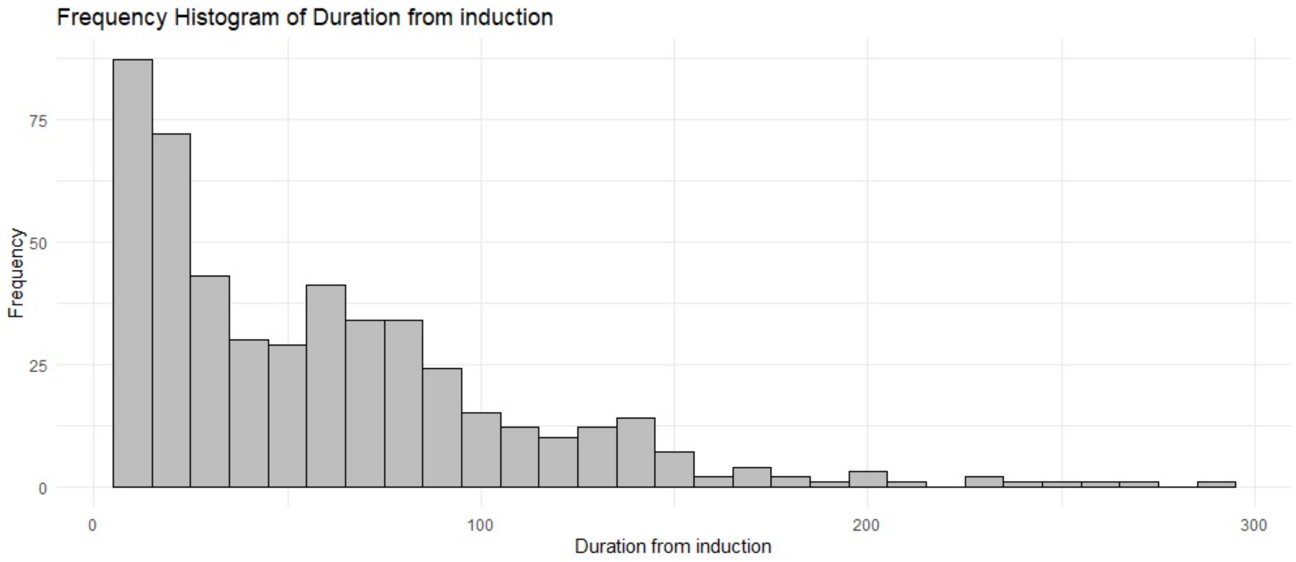

14 Characterization and effects of the inspiratory time

- n = 470
- Linear relationship between C_{dyn} and Inspiratory time:
  - p <0.001, Adjusted R^2 = 0.038

Supplementary Figure 13. Frequency histogram of the inspiratory time applied (ventilator setting, in minutes) at the time of determination of the dynamic compliance (C_{dyn}) in 470 dogs anaesthetized in 11 centres across six countries. Anaesthetic management was at the discretion of the local anaesthesia team, and the dogs were undergoing clinical procedures based on their individual conditions.

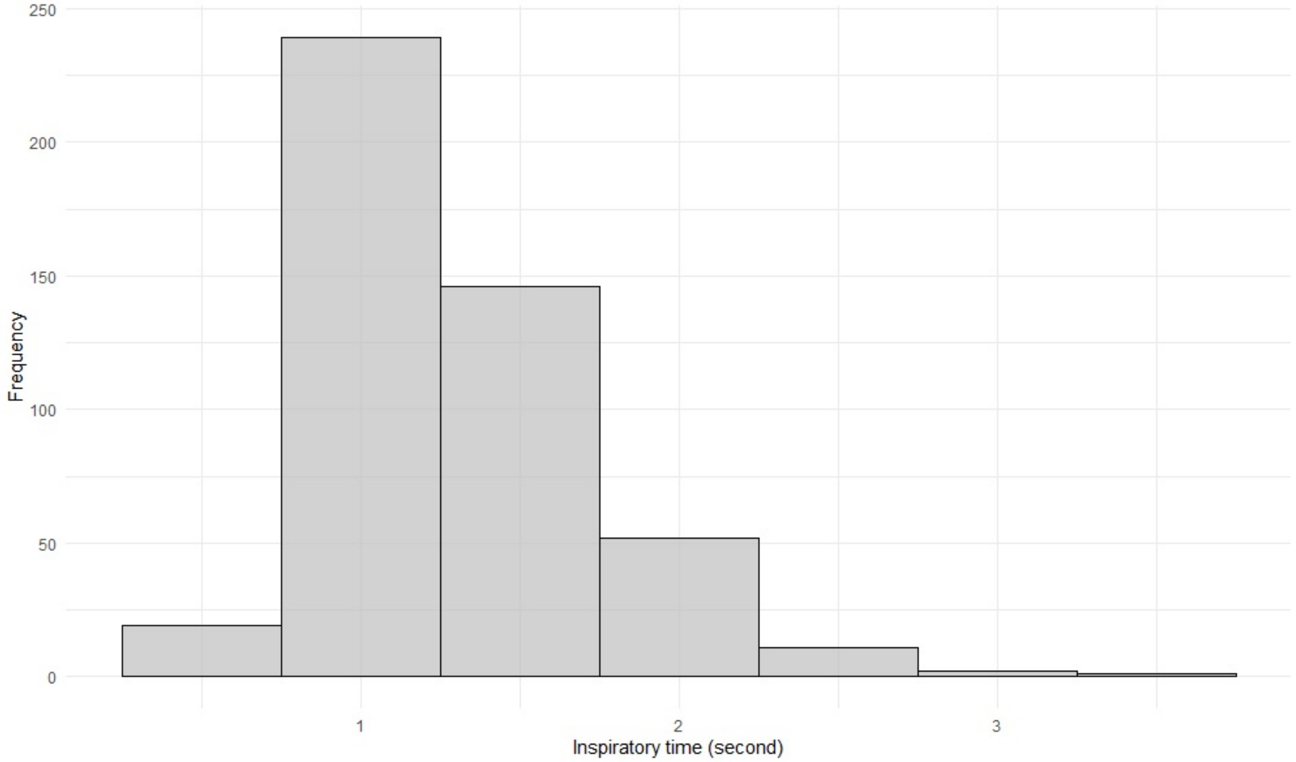

15 Characterization and effects of the ventilation mode

- n = 489
- Wilcoxon rank-sum test for C_{dyn} depending on the ventilation mode used (volume-controlled vs pressure-controlled ventilation):
  - *p*-value = 0.711

Supplementary Figure 14A. Frequency histogram of the ventilation modes use (volume-controlled ventilation vs pressure-controlled ventilation in 489 dogs anaesthetized in 11 centres across six countries. Anaesthetic management was at the discretion of the local anaesthesia team, and the dogs were undergoing clinical procedures based on their individual conditions.

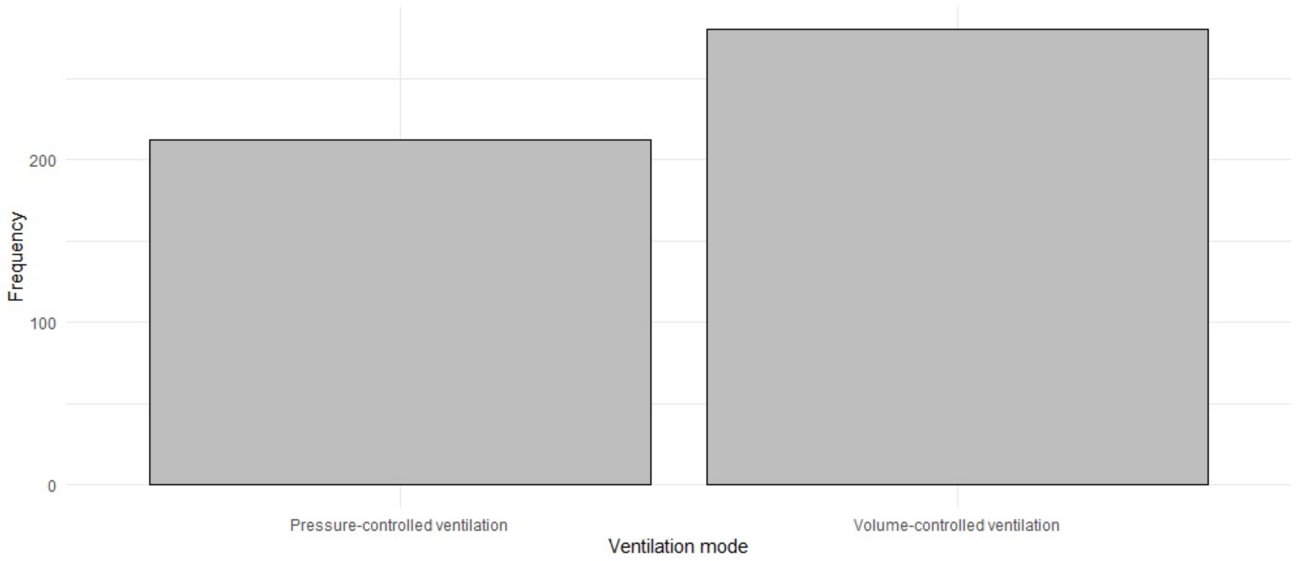

Supplementary Figure 14B. Box plots (median, interquartiles, range, outsiders) of dynamic compliance (C_{dyn}) according to the ventilation mode used (volume-controlled vs pressure-controlled ventilation) in 489 dogs anaesthetized in 11 centres across six countries. Anaesthetic management was at the discretion of the local anaesthesia team, and the dogs were undergoing clinical procedures based on their individual conditions.

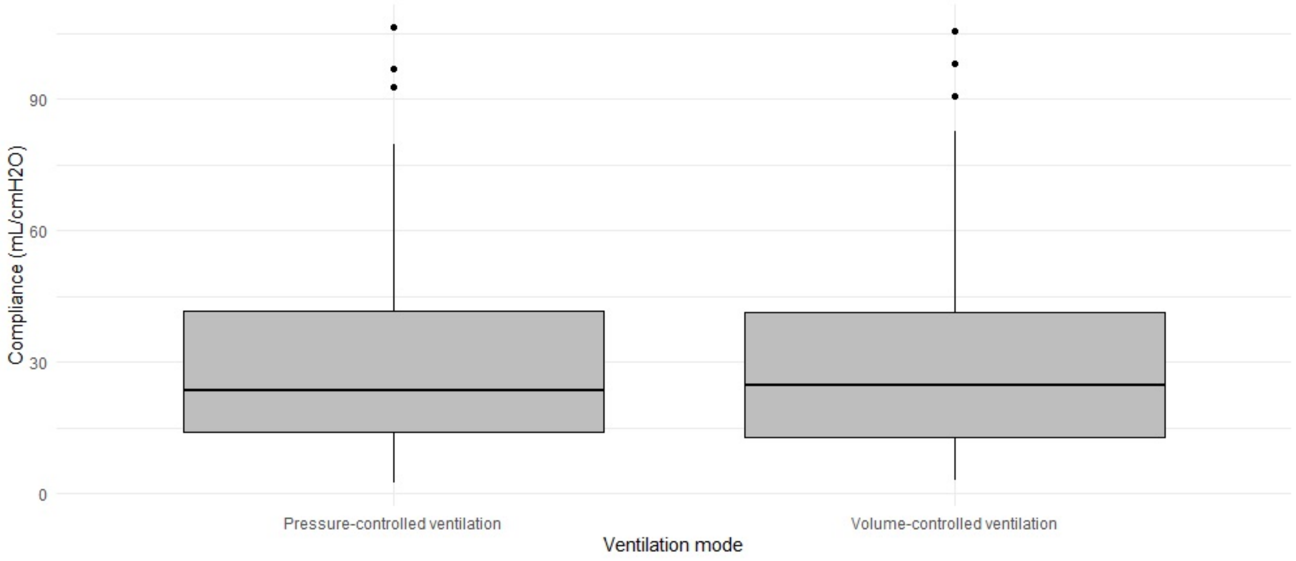

16 Characterization and effects of the Positive End Expiratory Pressure (PEEP)

- n = 481
- Linear relationship between C_{dyn} PEEP
  - p <0.001, Adjusted R^2 = 0.023
- No effect of PEEP inclusion in the C_{dyn}/Body mass model (p = 0.283)
  - Adjusted R^2 unchanged (0.654 -> 0.654)
  - AIC unchanged (3737.91 -> 3738.75)
  - BIC increased (3750.44 -> 3755.46)

Supplementary Figure 15A. Frequency histogram of the Positive End Expiratory Pressure (PEEP, in cmH_2O) set at the time of the measurements in 481 dogs anaesthetized in 11 centres across six countries. Anaesthetic management was at the discretion of the local anaesthesia team, and the dogs were undergoing clinical procedures based on their individual conditions.

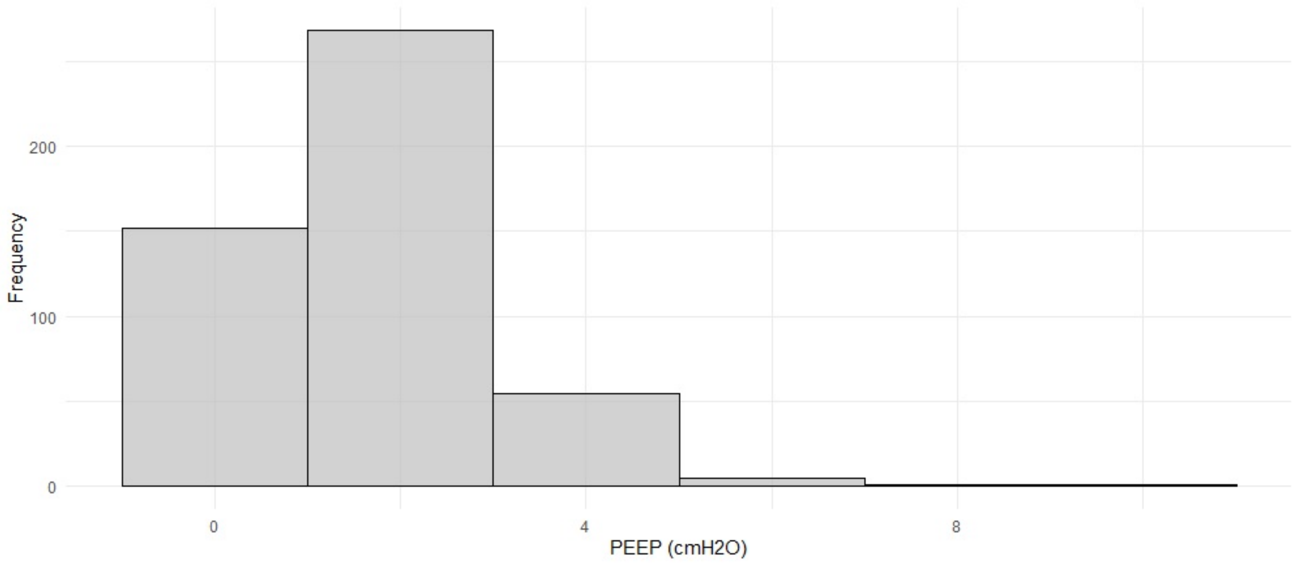

Supplementary Figure 15B. Best-fitting linear regression of C_{dyn} against the Positive End Expiratory Pressure (PEEP, in cmH_2O) set at the time of the measurements in a cohort of 481 dogs anaesthetized in 11 centres across six countries. Anaesthetic management was at the discretion of the local anaesthesia team, and the dogs were undergoing clinical procedures based on their individual conditions.

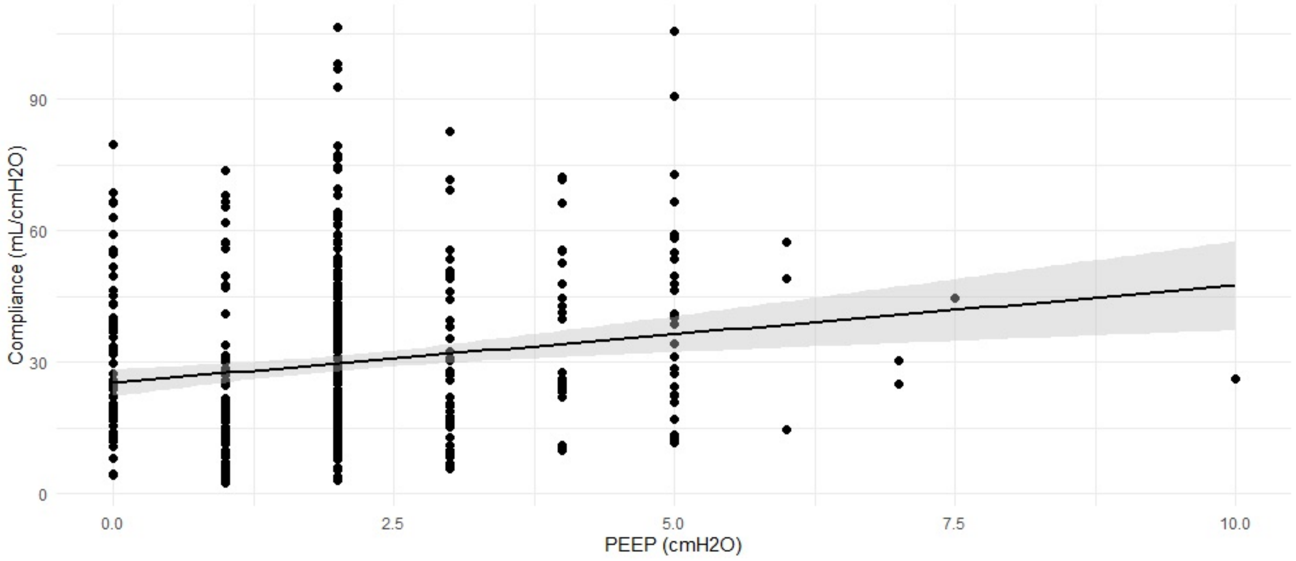

17 Development of the multiple linear regressions model

17.1 Variables considered in the final model:

- Body mass
- Internal diameter of the orotracheal tube (ETT_ID)
- Body Condition Score (BCS)
- Morphology
- Lateral recumbency
- Sensor used
- FIO_2 >80% for at least 10 minutes
- Time between induction and measurement
- Inspiratory time set
- Ventilation mode

17.2 Multiple linear regressions model using a stepwise forward selection and backward elimination technique

The variable with the highest *p*-value is removed and the model compared (ANOVA). If there is no significant difference or the model without is better, the variable is removed. If removal of the variable is maintained, re-introduction of previously removed variables is tested again.

**Variables (n = 452)**	***p*-values**	**#**
ETT_ID	<0.001	1
BCS	<0.001	2
Morphology	0.097	6
Lateral recumbency	0.194	8
Sensor	<0.001	4
FIO_2 >80% for at least 10 min.	0.032	5
Duration since induction	0.142	7
Inspiratory time	<0.001	3
Ventilation mode	0.909	9

a. Effect of the ventilation mode:

***p* = 0.909**	**Adjusted R^2**	**AIC**	**BIC**
With Ventilation mode	0.725	3408.109	3469.814
Without Ventilation mode	0.726	3406.122	3463.714

The ventilation mode is removed.

**Variables (n = 453)**	***p*-values**	**#**
ETT_ID	<0.001	1
BCS	<0.001	3
Inspiratory time	<0.001	2
Sensor	0.001	4
FIO_2 >80% for at least 10 min.	0.014	5
Morphology	0.091	6
Duration since induction	0.130	7
Lateral recumbency	0.191	8

b. Effect of the lateral recumbency:

p **= 0.191**	**Adjusted R^2**	**AIC**	**BIC**
With Lateral recumbency	0.726	3412.717	3470.34
Without Lateral recumbency	0.726	3412.479	3465.985

The lateral recumbency is removed.

**Variables (n = 453)**	***p*-values**	**#**
ETT_ID	<0.001	1
BCS	<0.001	3
Inspiratory time	<0.001	2
Sensor	0.001	4
FIO_2 >80% for at least 10 min.	0.019	5
Morphology	0.074	6
Duration since induction	0.165	7

c. Effect of the duration since anaesthesia induction:

p **= 0.165**	**Adjusted R^2**	**AIC**	**BIC**
With Duration	0.726	3412.479	3465.985
Without Duration	0.725	3412.461	3461.852

The time between induction of anaesthesia and the measurement is removed.

**Variables (n = 462)**	***p*-values**	**#**
ETT_ID	<0.001	1
BCS	<0.001	3
Inspiratory time	<0.001	2
Sensor	<0.001	4
FIO_2 >80% for at least 10 min.	0.014	5
Morphology	0.056	6

d. Effect of the morphology:

$p = 0.132$	Adjusted R^2	AIC	BIC
With Morphology	0.725	3480.121	3529.748
Without Morphology	0.725	3480.272	3521.628

The morphology is removed.

Variables (n = 462)	*p*-values	#
ETT_ID	<0.001	1
BCS	<0.001	4
Inspiratory time	<0.001	2
Sensor	<0.001	3
FIO_2 >80% for at least 10 min.	0.014	5

e. Effect of the FIO_2 >80% for at least 10 minutes before the measurement:

$p = 0.014$	Adjusted R^2	AIC	BIC
With FIO_2 >80% for at least 10 min.	0.724	3480.272	3521.628
Without FIO_2 >80% for at least 10 min.	0.720	3484.465	3521.685

The FIO_2 >80% for at least 10 minutes is kept in the model.
